# Phenformin activates ER stress to promote autophagic cell death via NIBAN1 and DDIT4 in oral squamous cell carcinoma independent of AMPK

**DOI:** 10.1038/s41368-024-00297-w

**Published:** 2024-05-08

**Authors:** Dexuan Zhuang, Shuangshuang Wang, Huiting Deng, Yuxin Shi, Chang Liu, Xue Leng, Qun Zhang, Fuxiang Bai, Bin Zheng, Jing Guo, Xunwei Wu

**Affiliations:** 1 School and Hospital of Stomatology, Cheeloo College of Medicine, Shandong University & Shandong Key Laboratory of Oral Tissue Regeneration & Shandong Engineering Research Center of Dental Materials and Oral Tissue Regeneration & Shandong Provincial Clinical Research Center for Oral Diseases, Jinan, China; 2 Engineering Laboratory for Biomaterials and Tissue Regeneration, Ningbo Stomatology Hospital, Savaid Stomatology School, Hangzhou Medical College, Ningbo, China; 3 Cedars-Sinai Cancer Institute, Department of Biomedical Sciences, Cedars-Sinai Medical Center, Los Angeles, CA USA

**Keywords:** Autophagy, Oral cancer

## Abstract

The efficient clinical treatment of oral squamous cell carcinoma (OSCC) is still a challenge that demands the development of effective new drugs. Phenformin has been shown to produce more potent anti-tumor activities than metformin on different tumors, however, not much is known about the influence of phenformin on OSCC cells. We found that phenformin suppresses OSCC cell proliferation, and promotes OSCC cell autophagy and apoptosis to significantly inhibit OSCC cell growth both in vivo and in vitro. RNA-seq analysis revealed that autophagy pathways were the main targets of phenformin and identified two new targets DDIT4 (DNA damage inducible transcript 4) and NIBAN1 (niban apoptosis regulator 1). We found that phenformin significantly induces the expression of both DDIT4 and NIBAN1 to promote OSCC autophagy. Further, the enhanced expression of DDIT4 and NIBAN1 elicited by phenformin was not blocked by the knockdown of AMPK but was suppressed by the knockdown of transcription factor ATF4 (activation transcription factor 4), which was induced by phenformin treatment in OSCC cells. Mechanistically, these results revealed that phenformin triggers endoplasmic reticulum (ER) stress to activate PERK (protein kinase R-like ER kinase), which phosphorylates the transitional initial factor eIF2, and the increased phosphorylation of eIF2 leads to the increased translation of ATF4. In summary, we discovered that phenformin induces its new targets DDIT4 and especially NIBAN1 to promote autophagic and apoptotic cell death to suppress OSCC cell growth. Our study supports the potential clinical utility of phenformin for OSCC treatment in the future.

## Introduction

OSCC is the most frequent type of head and neck cancer, and is the sixth most common type of cancer in the world^1^ with nearly 700 000 new cases and 380 000 deaths each year.^2^ In Asia, more than 90% of head and neck cancer cases are OSCC,^3^ which is also the most common cancer of the oral and maxillofacial region in China. At present, based on the pathological diagnosis and tumor stage, OSCC is mainly treated with a combination of surgery, chemotherapy, and radiotherapy.^4^ Despite the continuous innovation of therapeutic techniques, such as nano-drug delivery systems and bionic technology, the average mortality rate of OSCC patients has remained around 50% in recent years^5^ because of local recurrence and distant metastasis.^6,7^ This is likely due to the abundant blood flow in the oral and maxillofacial region and frequent movements of lips, tongue, and facial muscles. In addition, surviving OSCC patients have a high rate of malformations because of the ability of OSCC to damage the upper gastrointestinal tract and respiratory tract,^8^ which severely impacts survival and the quality of life of OSCC patients. Thus, the clinical treatment of OSCC remains a critical challenge.

Recent studies have demonstrated that cell autophagy has a crucial function in OSCC initiation and development.^9–11^ Autophagy can be categorized into three types according to the different pathways of cellular material translocation into lysosomes: macro-autophagy, chaperone-mediated autophagy, and micro-autophagy.^12^ The term “autophagy” usually refers to macro-autophagy, which is also the type most closely associated with cancer progression.^13^ The promotion of autophagic cell death has been shown to suppress the growth of various types of tumor cells including OSCC cells.^9,10,14^ For example, ursolic acid has been reported to promote apoptosis and autophagy by inhibiting the AKT/mTOR/NF-κB pathway and significantly inhibits the migration and invasion of the OSCC cell lines CA-922 and SCC-2095.^15^ Several studies have reported low expression levels of Beclin-1, an indicator of autophagy, in OSCC specimens,^16–18^ and silencing Beclin-1 significantly promotes OSCC migration, proliferation and invasion while inhibiting apoptosis.^19^ These results suggest that autophagy is closely related to the rapid development, distant metastasis, and poor prognosis of OSCC. Thus, targeting autophagy may provide a new therapeutic method for the clinical treatment of OSCC.

Phenformin, a derivative of metformin, was found to have broad-spectrum anti-tumor effects similar to metformin on various types of cancers, including malignant melanoma,^20,21^ breast cancer,^22^ and thyroid cancer.^23,24^ The accumulation of studies has demonstrated that phenformin produces much stronger anti-tumor effects compared to metformin for various types of tumors (e.g., breast cancer, prostate cancer, lung cancer, melanoma, and glioblastoma)^25^ due to its high lipophilicity and long half-life.^26^ Xia et al. reported that the combined application of metformin and nelfinavir (a broad-spectrum cancer drug) induces autophagy and sensitizes NK cells to exert anti-tumor effects in xenograft experiments with human cervical cancer cells and nude mouse cervical cancer cells.^27^ Chen et al. reported that metformin inhibits AR-negative prostate cancer growth by inducing tumor cell autophagy and blocking the cell cycle via the AMPK/autophagy pathway.^28^ Those results demonstrated that tumor cell death induced by metformin occurs through the promotion of autophagy formation in tumor cells, suggesting that as a derivative of metformin, phenformin may exert anti-cancer effects by inhibiting cell proliferation and promoting apoptosis, and that autophagy also plays an important role. In addition, we have reported that phenformin can inhibit OSCC cell growth,^29^ but the role of phenformin on OSCC needs documentation in detail, and especially the underlying mechanism(s) remains to be clarified. The purpose of the present study was to investigate whether phenformin can exert anti-OSCC effects through the autophagy pathway and to characterize the molecular mechanism involved.

## Results

### Phenformin significantly suppresses OSCC cell growth in vitro and in vivo

**Fig. 1:**
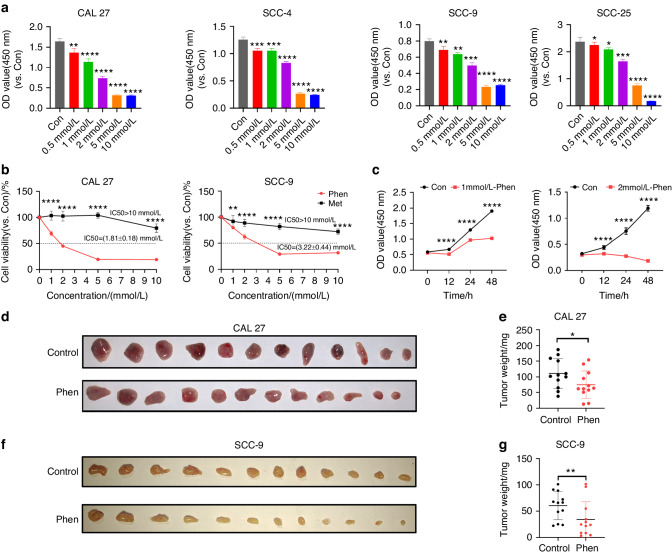
Phenformin suppresses OSCC cell growth in vitro and in vivo. **a** Four OSCC cell lines were treated with different concentrations of phenformin as indicated, or with PBS as a control. At 48 h, cells were collected and analyzed using the CCK-8 assay for cell viability. **b** CAL 27 cells and SCC-9 cells were treated with various concentrations of phenformin or metformin as indicated. At 48 h, cells were collected and analyzed using the CCK-8 assay for cell viability. **c** CAL 27 cells were treated with 1 mmol/L (left) or 2 mmol/L (right) phenformin as indicated, or with PBS as a control. Cells were collected at the different time points indicated and were analyzed using the CCK-8 assay for cell viability. **d**, **f** Photos of tumors isolated from three mice of each group after xenografting CAL 27 (**d**) and SCC-9 cells (**f**) combined with oral administration of phenformin (150 mg/kg) or PBS for 2 weeks. **e**, **g** Quantification of the average weight of tumors from **d** and **f**, respectively. All experiments were carried out three times, error bars represent means ± SD in each group; when compared with the corresponding control(Con) group, *P* values are indicated with “*”, *P < 0.05, **P < 0.01, ***P < 0.001, ****P < 0.000 1

To characterize the effects of phenformin on the growth of OSCC cells, four different OSCC cell lines (CAL 27, SCC-9, SCC-4, and SCC-25) were treated with different concentrations of phenformin. As shown in Fig. 1a, phenformin remarkably decreased the growth of all four different OSCC cell lines starting at a concentration of 0.5 mmol/L, and the inhibitory effect increased with increasing phenformin concentration. In order to conduct the study efficiently, we decided to randomly select two out of the four OSCC cell lines—CAL 27 and SCC-9—for all subsequent experiments. Next, we compared the effects of phenformin and metformin on OSCC growth, another well-known biguanide. First, we tested the effect of metformin on CAL 27 and SCC-9 cells at different concentrations, which showed that metformin inhibited OSCC cell growth at a concentration of at least 10 mmol/L, which was 20 times higher than that of phenformin (Fig. S1a, b). We then analyzed the IC50 of phenformin and metformin on CAL 27 and SCC-9 cells and found that the IC50 of phenformin is 1.81 mmol/L and 3.22 mmol/L on CAL 27 and SCC-9 OSCC cells, respectively, while the IC50 of metformin is higher than 10 mmol/L (Fig. 1b). Importantly, the observed IC50 of phenformin on OSCC cells was significantly lower than that on normal gingival epithelial cells (Fig. S1c). These data suggest that phenformin produces a more effective inhibition of OSCC cell growth compared to metformin. Therefore, we focused on investigating the effects of phenformin on the growth of OSCC cells in this study. To understand when the inhibitory effect on OSCC cells occurred after phenformin treatment, we treated OSCC cells with 1 or 2 mmol/L phenformin, and analyzed cell growth at different time points, which showed that phenformin inhibited cell growth within 12 h of treatment (Fig. 1c).

To further verify the inhibitory role of phenformin on the growth of OSCC cells, in vivo experiments were performed. CAL 27 and SCC-9 OSCC cells were subcutaneously injected into nude/nude mice. At 7 days, the nude mice were treated with 150 mg/kg phenformin or with PBS (control) by oral administration every day, and after 2 weeks of treatment, the tumors formed were collected (Fig. 1d, f). The size of tumors in the phenformin-treated group was smaller than those in the control group (Fig. 1d, f). Analysis of tumor weights revealed reduced tumor weights in the phenformin-treated mice (Fig. 1e, g). The mice fed with phenformin grew normally and they did not have significant weight changes after 2 weeks of treatment (Fig. S2a, b). As shown in Fig. S2c, no obvious pathological damage occurred in the heart, liver, or kidneys of mice treated with phenformin.

In sum, those results indicate that phenformin can significantly suppress OSCC cell growth both in vitro and in vivo.

### Phenformin suppresses OSCC growth by inhibiting OSCC cell proliferation and enhancing OSCC cell apoptosis and autophagy in vitro and in vivo

**Fig. 2:**
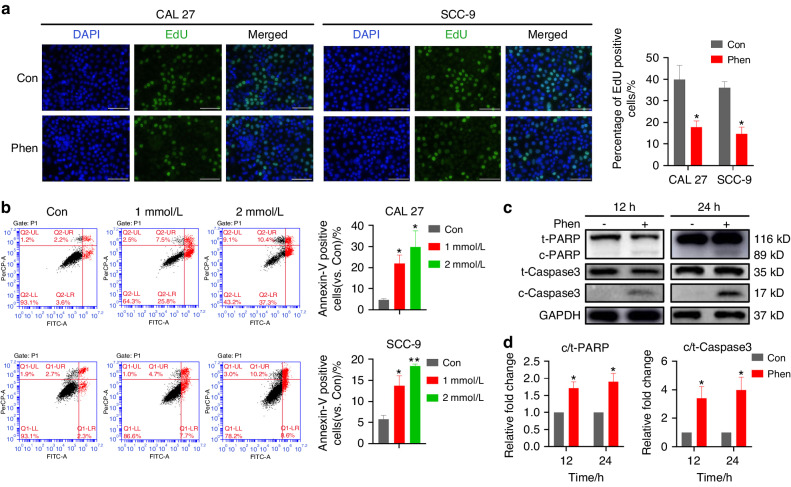
Phenformin blocks OSCC cell proliferation and promotes OSCC cell apoptosis in vitro. **a** Left: images of EdU staining (green) of CAL 27 and SCC-9 cells at 24 h after 1 mmol/L phenformin treatment. DAPI (blue) stain indicates nuclei. Scale bars = 200 μm. Right: quantification of EdU-positive cell percentage (%) of a total of 500 DAPI-positive cells counted. **b** Left: CAL 27 cells (upper row) and SCC-9 cells (lower row) were treated with 1 or 2 mmol/L phenformin for 24 h after which cells were collected and analyzed for apoptotic rate using FACS analysis. Right: quantification of apoptotic rate in images on the left. **c** Western blot analysis of total and cleaved forms of PARP (c-PARP) and Caspase 3 (c-Caspase 3) in CAL 27 cells treated with PBS (control) or with 1 mmmol/L phenformin for 12 and 24 h; GAPDH was used as a loading control. **d** Quantification of the relative levels of c-PARP and c-Caspase 3 in (**c**) normalized to the corresponding total protein bands (t-PARP or t-Caspase 3). All experiments were repeated three times, error bars represent means ± SD in each group; *P* values are indicated with “*” when compared to the corresponding control(Con) group, *P < 0.05, **P < 0.01

**Fig. 3:**
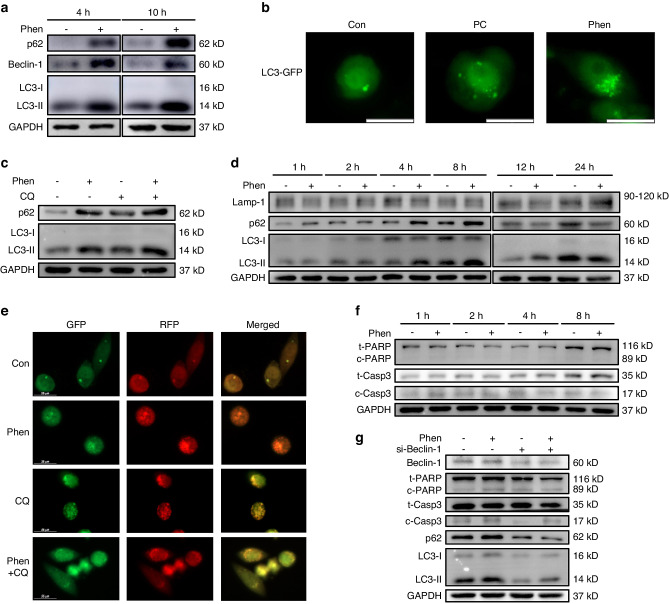
Phenformin promotes OSCC cell cytotoxic autophagy in vitro. **a** Western blot analysis of p62, Beclin-1, LC3-I, and LC3-II in CAL 27 cells treated with PBS (control) or with 1 mmol/L phenformin for 4 and 10 h; GAPDH was used as a loading control. **b** CAL 27 cells were infected with a LC3-GFP expression adenovirus (MOI = 100) for 24 h, and after infection, the cells were treated with 1 mmol/L phenformin or with PBS as a control for 6 h, after which the cells were checked for GFP expression using a fluorescence microscope. Scale bars = 20 μm. **c** Western blot analysis of p62, LC3-I, and LC3-II in CAL 27 cells treated with PBS (control) or 1 mmol/L phenformin or 10 μmol/L chloroquine (CQ) or 1 mmol/L phenformin plus 10 μmol/L chloroquine (Phen + CQ) for 12 h; GAPDH was used as a loading control. **d** Western blot analysis of Lamp-1, p62, and LC3 in CAL 27 cells treated without (−) or with (+) 1 mmol/L phenformin at different time points as indicated; GAPDH was used as a loading control. **e** Representative fluorescent images of CAL 27 cells transfected with a GFP-RFP-LC3 plasmid, followed by treatment with PBS (Con) or 1 mmol/L phenformin or 10 μmol/L chloroquine (CQ) or 1 mmol/L phenformin plus 10 μmol/L chloroquine (Phen + CQ) for 24 h. Scale bars = 25 μm. **f** Western blot analysis of apoptotic markers in CAL 27 cells treated without (−) or with (+) 1 mmol/L phenformin at different time points as indicated; GAPDH was used as a loading control. **g** Western blot analysis of apoptotic and autophagic markers and Beclin-1 in CAL 27 cells transfected with scramble siRNAs or Beclin-1 siRNAs with or without 1 mmol/L phenformin at 24 h and incubated with 1 mmol/L phenformin or PBS as a control for 12 h; GAPDH was used as a loading control. All experiments were repeated three times

In order to understand how phenformin suppresses OSCC cell growth, we then investigated the effects of phenformin on OSCC cell proliferation and cell death. First, in vitro assays were performed. The EdU proliferation assay was performed to test the effect of phenformin on the proliferation of OSCC cells, and showed reduced numbers of EdU-positive cells in the phenformin treatment group (Fig. 2a), indicating that treatment with phenformin reduced OSCC cell proliferation in vitro. Next, we investigated whether phenformin suppressed OSCC cell growth through the induction of cell death either by apoptosis or by autophagy, the two major mechanisms of cell death in vitro. Flow cytometry was performed on CAL 27 and SCC-9 OSCC cells treated with 1 or 2 mmol/L phenformin, and the results showed that phenformin treatment clearly enhanced the apoptotic rate in a concentration-dependent manner (Fig. 2b). The cell apoptosis induced by phenformin was further verified by Western blot analysis of the caspase pathways, which are activated in cells undergoing apoptosis. The results showed that levels of cleaved-caspase 3 (c-Caspase 3) and its target protein PARP (cleaved PARP, c-PARP) increased in OSCC cells at 12 and 24 h after phenformin treatment (Fig. 2c, d). To test whether phenformin could induce autophagy to promote OSCC cell death in vitro, both CAL 27 and SCC-9 OSCC cells treated with 1 mmol/L phenformin were collected at different time points (Fig. S3a, b) for qRT-PCR analysis to detect the expression levels of autophagic markers. PCR analysis revealed that the mRNA levels of *p62*, *ATG7*, *Beclin-1*, *ATG12*, and *Lamp-1* were significantly increased in OSCC cells at 6 h after phenformin treatment (Fig. S3a, b). The protein expression levels of Beclin-1, p62, and LC3-II were also elevated at 4 h after treatment with 1 mmol/L phenformin (Fig. 3a and Fig. S3c). Moreover, OSCC cells transfected with GFP labeled LC3, which labels autophagic cells, and then treated with phenformin significantly increased dot staining of GFP cells (Fig. 3b and Fig. S3d, e). These data suggest that phenformin can promote OSCC autophagy.

To explore whether phenformin induces autophagy through an increase in autophagosome formation or the inhibition of autophagic flux resulting in autophagosome accumulation, we first used chloroquine (CQ), which blocks autophagosome–lysosome fusion to inhibit autophagic flux. Combining CQ with or without phenformin to treat OSCCs, we observed that both phenformin and CQ could increase the expression level of p62 and LC3-II, and the combination of phenformin and CQ (phen + CQ) further elevated the levels of LC3-II and p62 (Fig. 3c and Fig. S3f), which suggested that phenformin likely promotes autophagosome formation.

To further confirm that phenformin promotes OSCC autophagy mainly due to the induction of autophagosome formation, we conducted a time-course analysis of Lamp-1 (a lysosomal marker) and p62 (an autophagosome marker) protein levels in phenformin-treated cells. We observed an increase in p62 as well as LC3-II levels that occurred at early time points (up to 12 h) and a subsequent significant decrease of p62 at a late time point (24 h) in phenformin-treated cells compared to the control cells. In contrast, the Lamp-1 level did not increase at early time points, but showed a clear increase at 24 h after phenformin treatment (Fig. 3d and Fig. S3g). Additionally, we transfected CAL 27 cells with a GFP-RFP-LC3 plasmid to mark autophagosomes during the treatment with phenformin. As reported, after fusion with lysosomes, the green signals (GFP) decay due to acidic conditions, while the red signals (RFP) remain relatively stable.^30^ We found that at 24 h after treatment the red signals, but not the green signals were more pronounced in phenformin-treated cells, while stronger yellow signals were observed in CQ-treated, especially in Phen + CQ-treated cells, when compared to the control cells (Fig. 3e and Fig. S3h). This result further confirmed that phenformin did not significantly block the autophagic flux. Taking these data together, we conclude that phenformin promotes OSCC cell autophagy mainly through the induction of autophagosome formation.

In our above experiments, we observed that the induction of autophagy by phenformin at 4 h after treatment (Fig. 3a), occurred earlier than the increased apoptosis observed at 12 h after treatment (Fig. 2c). To further confirm that observation, we performed Western blot analysis of both autophagic and apoptotic markers on the same cells treated with phenformin for 1, 2, 4, and 8 h. Indeed, we found that the increased expression of autophagic markers appeared at 4 h (Fig. 3d and Fig. S3g) while the expression of apoptotic markers was still not increased at 8 h (Fig. 3f and Fig. S3i).

It has been previously shown that autophagy can induce apoptosis, and therefore we investigated whether the autophagy induced by phenformin can promote apoptosis. Knockdown of the autophagic gene Beclin-1 not only blocked the induction of autophagy, but also reduced the expression of apoptotic markers induced by phenformin (Fig. 3g and Fig. S3j), suggesting that phenformin promotes OSCC apoptosis through the induction of autophagy. Finally, we found that the knockdown of Beclin-1 could rescue OSCC growth inhibition induced by phenformin (Fig. S4), indicating that the induction of autophagy by phenformin can enhance OSCC cell death in vitro.

**Fig. 4:**
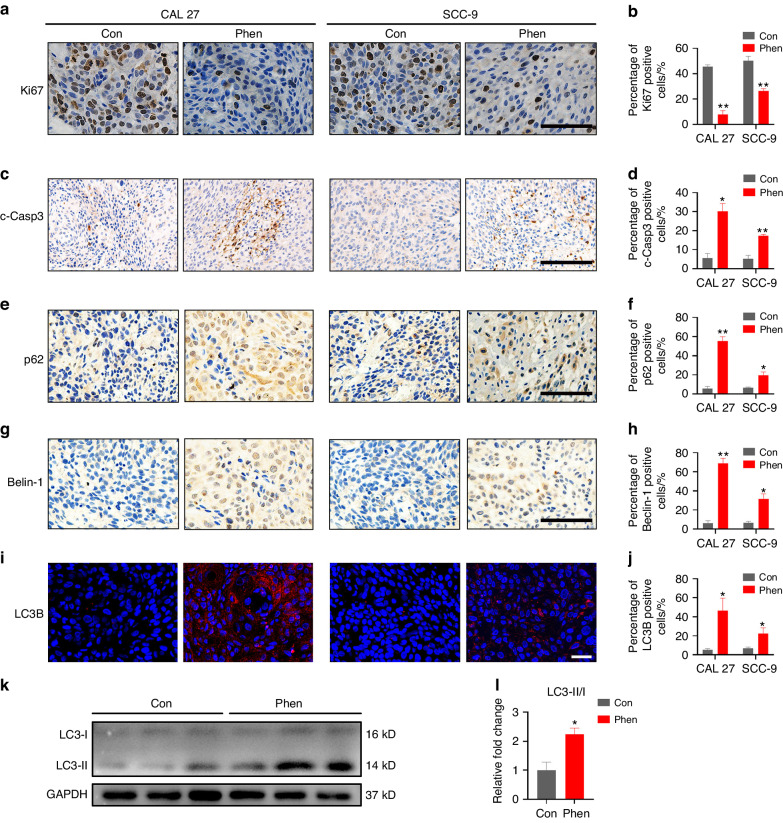
Phenformin inhibits OSCC cell proliferation and enhances OSCC cell apoptosis and autophagy in vivo. Representative images of immunohistochemical (**a**, **c**, **e**, **g**) and immunofluorescence staining (**i**) of tumors from PBS (control) or phenformin-treated mice with xenografted CAL 27 (left) and SCC-9 cells (right) to detect Ki67 (**a**), c-Caspase 3 (**c**), p62 (**e**), Beclin-1 (**g**), and LC3B (**i**) expression (red or brown). DAPI (blue) staining identifies nuclei. Scale bars in **a**, **e**, **g**: 200 μm; in **c**: 500 μm; in **i**: 50 μm. **b**, **d**, **f**, **h**, **j** Quantification of Ki67 (**b**), c-Caspase 3 (**d**), p62 (**f**), Beclin-1 (**h**), and LC3B (**j**) positive cell percentage (%) of a total of 500 DAPI-positive cells. **k** Western blot analysis of LC3-I and LC3-II in three independent tumors from PBS (control) or phenformin-treated mice; **l** Quantification of the relative expression level of LC3-II was normalized to LC3-I in (**k**). All experiments were repeated three times, error bars represent means ± SD in each group; *P* values are indicated with “*”, *P < 0.05, **P < 0.01

In order to verify the vitro results, the tumors derived from mice shown in Fig. 1d, f were analyzed for their status of OSCC cell proliferation, apoptosis, and autophagy in vivo. First, the tumors were stained for the proliferation marker Ki67, which revealed significantly fewer numbers of Ki67-positive cells in tumors treated with phenformin (Fig. 4a, b). The immunostaining of cleaved-caspase 3 (c-Casp3) in OSCC tumors indicated that there were increased numbers of apoptotic cells in the tumors of mice treated with phenformin (Fig. 4c, d). Finally, the OSCC tumors shown in Fig. 1d, f were stained for the autophagic markers p62, Beclin-1, and LC3B, and the numbers of positive cells were significantly increased in tumors of mice treated with phenformin (Fig. 4e–j), and the protein expression level of LC3-II was also elevated as well in phenformin-treated tumors (Fig. 4k, l). These data suggested that phenformin can inhibit OSCC cell proliferation and induce OSCC cell autophagy and apoptosis in vivo. Taken together, we concluded that phenformin not only inhibits OSCC cell proliferation, but also promotes OSCC cell apoptosis and autophagy, which suppresses OSCC cell growth in vitro and in vivo.

### RNA-seq analysis reveals that phenformin mainly induces autophagy in OSCC cells associated with the induced expression of DDIT4 and NIBAN1

**Fig. 5:**
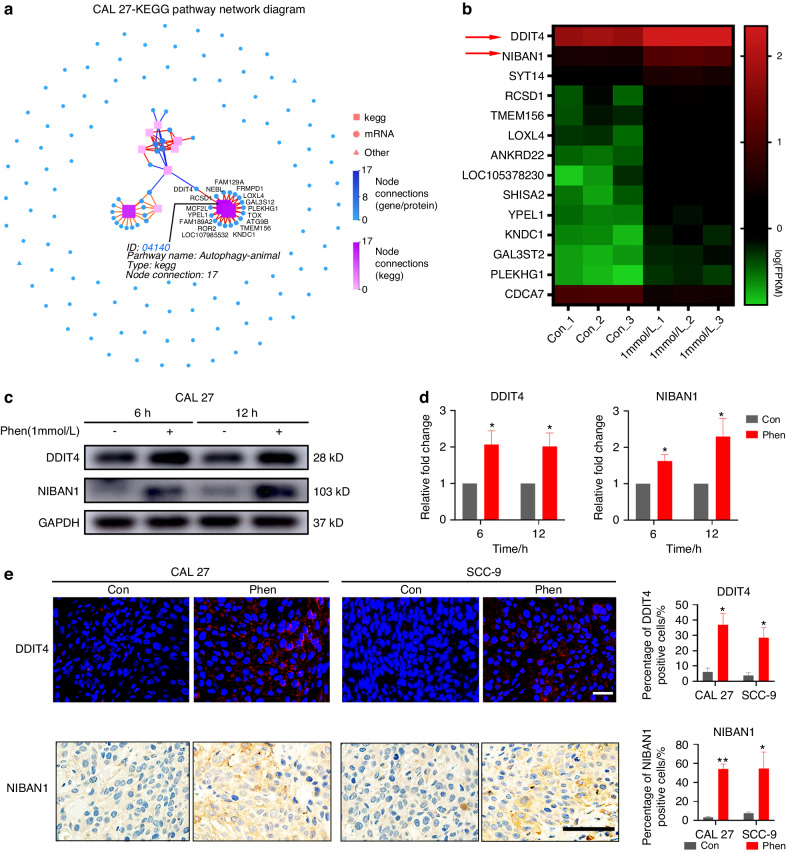
Phenformin mainly regulates the autophagic signaling pathway and induces expression of the autophagy-related genes *DDIT4* and *NIBAN1*. **a** KEGG Pathway Network Diagram of DEGs in CAL 27 cells. Blue circles and red squares represent different mRNAs and the KEGG pathway, respectively. The darker the color represents the more mRNAs or the KEGG pathway were connected to the pathway or mRNA. **b** Heat map analysis of the 14 most significant DEGs (*q* value < 0.05) related to the autophagy signaling pathway from both CAL 27 and SCC-9 cells treated with or without 1 mmol/L phenformin based on the FPKM value of each sample. The *X* axis represents the different samples, whereas the *Y* axis represents DEGs. The color (from green to red) represents DEG expression intensities from low to high. **c** Western blot analysis of DDIT4 and NIBAN1 in CAL 27 cells treated with PBS (control) or with 1 mmol/L phenformin for 6 and 12 h; GAPDH was used as a loading control. **d** Quantification of the relative levels of DDIT4 and NIBAN1 normalized to the GAPDH band. **e** Left: representative images of immunofluorescence staining (DDIT4, top row) and immunohistochemical staining (NIBAN1, bottom row) of tumors from mice xenografted with CAL 27 (left) and SCC-9 cells (right) to detect DDIT4 (upper row) and NIBAN1 (lower row) expression (DDIT4: red, NIBAN1: brown). DAPI (blue) staining identifies nuclei. Scale bars of DDIT4: 50 μm, NIBAN1: 200 μm. Right: quantification of DDIT4 or NIBAN1-positive cell percentage (%) of a total of 500 DAPI-positive cells. All experiments were repeated three times (**c**–**e**), error bars represent means ± SD in each group; *P* values are indicated with “*”, *P < 0.05, **P < 0.01

To further understand what major signaling pathways are regulated by phenformin to suppress OSCC cell growth, we performed RNA-seq analysis of global transcriptome alterations of CAL 27 OSCC cells treated with or without 1 mmol/L phenformin for 12 h. There were 135 upregulated genes and 8 downregulated genes in CAL 27 OSCC cells treated with phenformin compared with the untreated control group. The distribution of upregulated differentially expressed genes (DEGs) (red dots) and downregulated DEGs (green dots) between PBS (as a control) and phenformin-treated cells is shown in Fig. S5a. The enriched KEGG pathways were analyzed, and the diagram of pathway interaction networks is presented in Fig. 5a. The autophagy-related pathway was the most significantly enriched pathway in phenformin-treated CAL 27 OSCC cells. After that, we also performed RNA sequencing of SCC-9 cells treated with phenformin or PBS as a control, and 49 upregulated DEGs and 16 downregulated DEGs were screened in Volcano plots (Fig. S5a). Interestingly, the autophagy-related pathway was also the most significantly enriched KEGG pathway in SCC-9 cells treated with phenformin (Fig. S5b). Heat map analysis showed that the 14 most significant DEGs were related to the autophagy signaling pathway between the control and phenformin-treated OSCC cells (Fig. 5b), and, based on their expression levels, the ranking top 2 genes were *DDIT4* and *NIBAN1* (Fig. 5b). *DDIT4*, DNA damage inducible transcript 4, also known as *REDD1*, has been reported to regulate cell growth, proliferation, and survival.^31,32^ *NIBAN1*, niban apoptosis regulator 1, has been reported to regulate the phosphorylation of many proteins involved in transcription regulation.^33^ DDIT4 (REDD1) has been reported to be induced by metformin, but no study has reported the effects of biguanides (either metformin or phenformin) on NIBAN1 expression.^34^ qRT-PCR analysis verified that phenformin treatment significantly increased the expression of *DDIT4* and *NIBAN1* both in CAL 27 and in SCC-9 cells (Fig. S5c, d), which was confirmed by Western blot analysis of DDIT4 and NIBAN1 protein levels (Fig. 5c, d). Importantly, the increased expression of DDIT4 and NIBAN1 was also observed in OSCC tumors treated with phenformin (Fig. 5e). Taken together, RNA-seq analysis revealed that activation of the autophagy signaling pathway was the major effect of phenformin on OSCC cells and RNA-seq analysis identified two important genes, *DDIT4* and *NIBAN1*, which were significantly induced by phenformin treatment.

### Phenformin induces autophagy formation through the regulation of DDIT4 and NIBAN1 expression

**Fig. 6:**
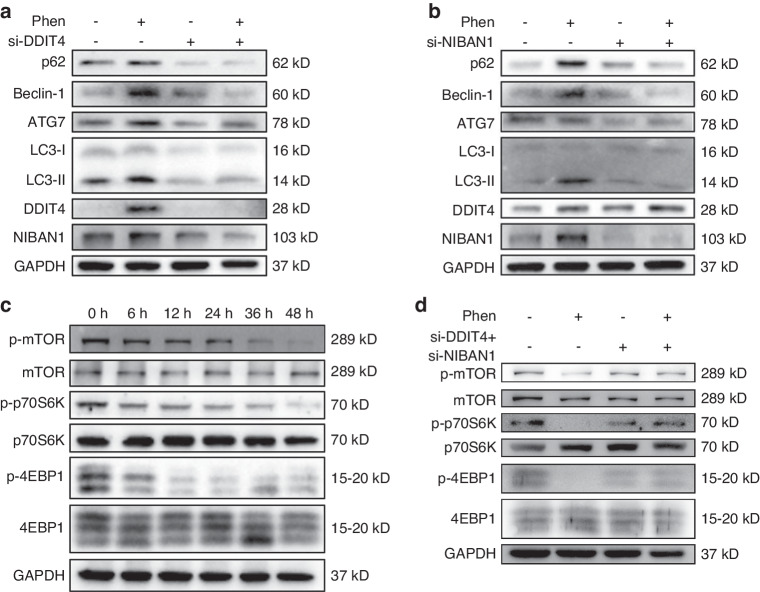
Inhibition of DDIT4 and NIBAN1 expression blocks phenformin-induced autophagy. **a**, **b** Expression of DDIT4, NIBAN1, p62, Beclin-1, ATG7, and LC3-I/II analyzed by Western blot in CAL 27 cells transfected with DDIT4 siRNA (si-DDIT4) (**a**) or NIBAN1 siRNA (si-NIBAN1) (**b**) or the corresponding controls (NC) at 12 h after treatment with 1 mmol/L phenformin (Phen) or with PBS as a control. The quantification of protein bands is shown in Fig. S6b, d. **c** Western blot analysis of total and phosphorylated mTOR, p70S6K, and 4EBP1 in CAL 27 cells treated with PBS (0 h, control) or with 1 mmol/L phenformin for 6, 12, 24, 36, and 48 h; GAPDH was used as a loading control and the quantification of protein bands is shown in Fig. S7a. **d** Western blot analysis of total and phosphorylated mTOR, p70S6K, and 4EBP1 in CAL 27 cells transfected with both si-DDIT4 and si-NIBAN1 or the corresponding controls (NC) at 12 h after treatment with 1 mmol/L phenformin (Phen) or with PBS as a control; GAPDH was used as a loading control and the quantification of protein bands is shown in Fig. S7b. All experiments were repeated three times

Next, to determine whether phenformin promotes autophagy through the high expression levels of DDIT4 and/or NIBAN1, OSCC cells were transfected with DDIT4 or NIBAN1 siRNAs or a control siRNA with or without 1 mmol/L phenformin. The expression level of DDIT4 for both mRNA (Fig. S6a) and protein (Fig. 6a and Fig. S6b) was significantly decreased by the transfection of DDIT4 siRNA. The expression of p62, Beclin-1, ATG7, and LC3-II induced by phenformin was significantly suppressed by the knockdown of DDIT4 (Fig. 6a and Fig. S6a, b). The same experiment was then performed using transfection of a NIBAN1 siRNA. OSCC cells were transfected with NIBAN1 siRNA or a control siRNA with or without 1 mM phenformin, and the knockdown efficiency was confirmed by qRT-PCR analysis (Fig. S6c) and Western blot analysis (Fig. 6b and Fig. S6d). The expression of p62, Beclin-1, ATG7, and LC3-II induced by phenformin was also significantly suppressed by the knockdown of NIBAN1 (Fig. 6b and Fig. S6c, d). In addition, we obtained a similar result after the double knockdown of both DDIT4 and NIBAN1 (Fig. S6e). These data suggested that phenformin promotes autophagy depending on the regulation of DDIT4 and NIBAN1 expression.

DDIT4 has been reported to negatively regulate the mTOR pathway, and NIBAN1 has been shown to interact with the mTOR pathway to regulate apoptosis and autophagy.^35,36^ To confirm that, we observed that phenformin significantly reduced the phosphorylation level of mTOR and its downstream targets p70S6K and 4EBP1, which indicates that phenformin inhibits the mTOR pathway in OSCC cells (Fig. 6c and Fig. S7a). To investigate if the phenformin inhibition of the mTOR pathway was through the induction of DDIT4 and NIBAN1, OSCC cells were transfected with siRNAs targeting DDIT4 and NIBAN1, followed by treatment with phenformin. We found that the double knockdown of DDIT4 and NIBAN1 could rescue the inhibition of mTOR activation by phenformin (Fig. 6d and Fig. S7b). In addition, MHY1485, a mTOR activator, was used to treat OSCC cells treated with phenformin and the promotion of autophagy markers by phenformin was inhibited by MHY1485 (Fig. S7c). The above results suggest that phenformin induces DDIT4 and NIBAN1 expression to promote autophagy by inhibiting the mTOR signaling pathway.

### Phenformin controls DDIT4 and NIBAN1 expression through the positive regulation of ATF4 expression independent of AMPK activation

**Fig. 7:**
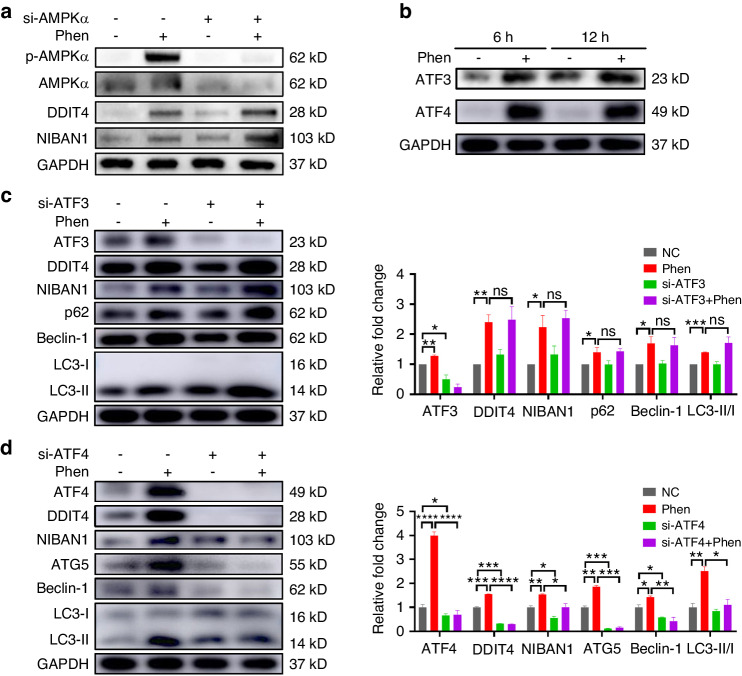
Phenformin induces ATF4 expression to control DDIT4 and NIBAN1 expression in OSCC cells. **a** Expression levels of AMPKα (AMPKα1 and AMPKα2), DDIT4, and NIBAN1 analyzed by Western blot in CAL 27 cells transfected with AMPKα1 siRNA plus AMPKα2 siRNA or with the corresponding controls (NC) at 12 h after treatment with 1 mM phenformin (Phen) or with PBS as a control. Quantification of protein bands is shown in Figure S8b. **b** Expression of ATF3 and ATF4 analyzed by Western blot in CAL 27 cells at 6 and 12 h after treatment with 1 mmol/L phenformin (Phen) or with PBS as a control. Quantification of protein bands is shown in Figure S9c. **c** Expression of ATF3, DDIT4, NIBAN1, p62, Beclin-1, and LC3-I/II analyzed by Western blot in CAL 27 cells transfected with ATF3 siRNA (si-ATF3) or corresponding controls (NC) at 12 h after treatment with 1 mmol/L phenformin (Phen) or with PBS as a control. Quantification of LC3-II was normalized to LC3-I and quantification of the other protein bands was normalized to GAPDH. **d** Expression of ATF4, DDIT4, NIBAN1, ATG5, Beclin-1, and LC3-I/II analyzed by Western blot in CAL 27 cells transfected with ATF4 siRNA (si-ATF4) or corresponding controls (NC) at 12 h after treatment with 1 mmol/L phenformin (Phen) or with PBS as a control. Quantification of LC3-II was normalized to LC3-I and quantification of the other protein bands was normalized to GAPDH. All experiments were repeated three times, error bars represent means ± SD in each group; *P* values are indicated with “*”, *P < 0.05, **P < 0.01, ***P < 0.001, ****P < 0.000 1

We then asked how phenformin regulates the expression of DDIT4 and NIBAN1 in OSCC cells. As phenformin is an AMPK activator, we checked whether phenformin activates AMPK to control DDIT4 and/or NIBAN1 expression. The knockdown of AMPK by transfection of both AMPK α1 and α2 siRNAs efficiently decreased AMPK expression (Fig. S8a), and AMPK inhibition did not block the induction of *DDIT4* or *NIBAN1* by phenformin treatment (Fig. S8a). This result was also confirmed by Western blot analysis (Fig. 7a and Fig. S8b). These results suggest that phenformin controls the expression of DDIT4 and NIBAN1 likely independent of AMPK activation. A previous study has shown that the transcription factor ATF4 controls the expression of DDIT4,^37,38^ therefore we analyzed the RNA-seq data from Fig. 5 and found that there were higher expression levels of *ATF3* and *ATF4* in the phenformin-treated OSCC cells (Fig. S9a). Both qRT-PCR and Western blot analysis of OSCC cells treated with 1 mmol/L phenformin confirmed that phenformin treatment significantly increased the expression of both ATF3 and ATF4 (Fig. 7b and Fig. S9b, c). To determine whether phenformin induces DDIT4 or NIBAN1 expression through the regulation of ATF expression, we first transfected siRNAs of ATF3 or ATF4 to knockdown the expression of ATF3 or ATF4 in OSCC cells with or without treatment by phenformin. The knockdown efficiency was confirmed by qRT-PCR analysis of *ATF3* or *ATF4* expression (Fig. S10). Further, we observed that the increased expression of *DDIT4* and *NIBAN1* together with autophagic markers elicited by phenformin treatment was not affected by the knockdown of ATF3 (Fig. S10a), but was significantly abrogated by the knockdown of ATF4 (Fig. S10b). These results were further confirmed by Western blot analysis of the protein levels of these genes (Fig. 7c, d). Taken together, these results suggest that phenformin promotes DDIT4 and NIBAN1 expression to induce autophagy through the positive regulation of ATF4, but not ATF3, and the regulation was independent of AMPK activation.

### Phenformin directly induces ER stress to control the expression of ATF4-DDIT4 and NIBAN1

**Fig. 8:**
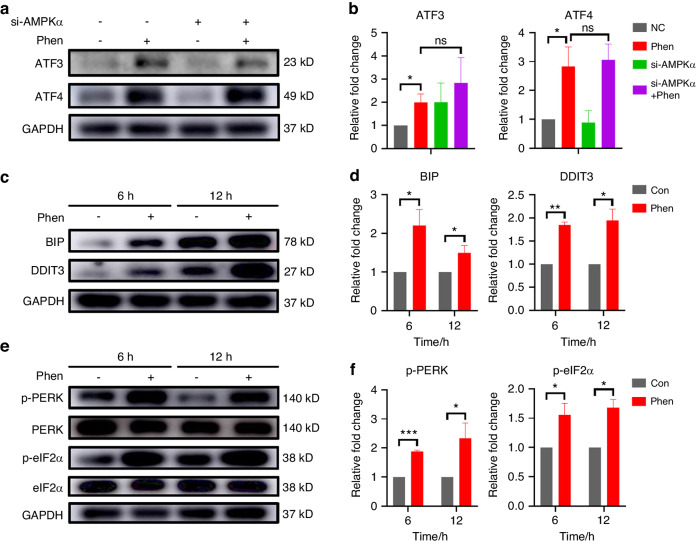
Phenformin induces ER stress in OSCC cells. **a** Expression levels of ATF3 and ATF4 analyzed by Western blot in CAL 27 cells transfected with AMPKα1 siRNA and AMPKα2 siRNA or corresponding controls (NC) at 12 h after treatment with 1 mmol/L phenformin (Phen) or with PBS as a control. **b** Quantification of protein bands was normalized to GAPDH. **c** Expression of BIP and DDIT3 analyzed by Western blot in CAL 27 cells at 6 and 12 h after treatment with 1 mmol/L phenformin (Phen) or with PBS as a control. **d** Quantification of the relative levels of BIP and DDIT3 was normalized to GAPDH. **e** Expression of total and phosphorylated PERK and eIF2α analyzed by Western blot in CAL 27 cells at 6 and 12 h after treatment with 1 mmol/L phenformin (Phen) or with PBS as a control. **f** Quantification of the relative levels of p-PERK and p-eIF2α was normalized to the corresponding total protein band. All experiments were repeated three times, error bars represent means ± SD in each group; *P* values are indicated with “*”, *P < 0.05, **P < 0.01, ***P < 0.001

Next, we confirmed that the knockdown of AMPK did not suppress the ATF3 or ATF4 expression induced by phenformin treatment (Fig. 8a, b and Fig. S11a). *ATF4*, a stress response gene, has been well-known to be selectively induced at the translational level by the activation of eukaryotic translation factor-2 alpha (eIF2α) during ER stress, which is activated by a number of cellular stresses such as hypoxia, metabolic stress, and so on.^39,40^ Metformin has been shown to activate ER stress by increasing the expression of BIP, DDIT3, and caspase-12 in vitro. Therefore, we hypothesized that phenformin increases ATF4 expression directly through the activation of ER stress. To confirm that hypothesis, we first checked the expression of BIP, DDIT3, and XBP1, which are associated with the activation of ER stress. We found that the expression of DDIT3 and XBP1 at 6 h was significantly induced by 1 mmol/L phenformin treatment in OSCC cells (Fig. 8c, d and Fig. S11b). The activation of ER stress results in increasing PERK activity to phosphorylate eIF2α, and we found that phenformin treatment clearly increased the phosphorylation level of both PERK and elF2α, along with the increased expression of ATF4 (Fig. 8e, f). These data suggested that phenformin can induce ER stress to activate PERK, and the activation of PERK results in the activation of its downstream target eIF2α, which increases the translation of ATF4.

**Fig. 9:**
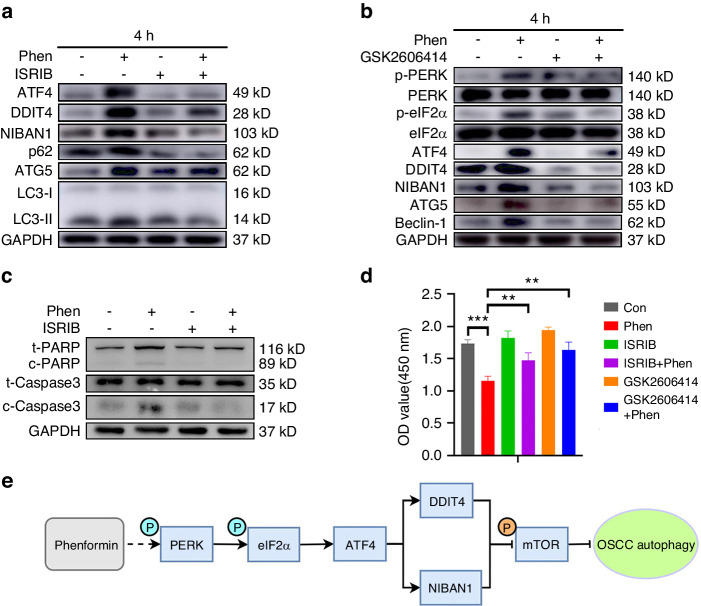
Phenformin induces the expression of ATF4, DDIT4, and NIBAN1 through the regulation of ER stress. **a** Protein expression levels of ATF4, DDIT4, NIBAN1, p62, ATG5, and LC3-I/II analyzed by Western blot in CAL 27 cells at 4 h after treatment with 1 mmol/L phenformin (Phen) or 200 nmol/L ISRIB or DMSO as a control. The quantification of protein bands is shown in Fig. S11d. **b** Protein expression levels of total and phosphorylated PERK and eIF2α, ATF4, DDIT4, NIBAN1, ATG5, and Beclin-1 analyzed by Western blot in CAL 27 cells at 4 h after treatment with 1 mmol/L phenformin (Phen) or with 1 μM GSK2606414 or DMSO as a control. The quantification of protein bands is shown in Fig. S11e. **c** Western blot analysis of total and cleaved forms of PARP (c-PARP) and Caspase 3 (c-Caspase 3) in CAL 27 cells at 12 h after treatment with 1 mmol/L phenformin (Phen) or 200 nmol/L ISRIB or DMSO as a control; GAPDH was used as a loading control. The quantification of protein bands is shown in Fig. S11f. **d** CAL 27 cells were treated with 1 mmol/L phenformin (Phen) or with 200 nmol/L ISRIB or 1 μmol/L GSK2606414 or DMSO as a control. At 24 h, cells were collected and analyzed by the CCK-8 assay for cell viability. **e** Scheme showing that phenformin activates PERK/eIF2α to promote autophagy by increasing ATF4 to induce DDIT4 and NIBAN1 expression. All experiments were repeated three times, error bars represent means ± SD in each group; *P* values are indicated with “*”, **P < 0.01, ***P < 0.001

To further verify the induction of ER stress by phenformin treatment to control ATF4 expression, we treated OSCC cells with phenformin together with a small-molecule ISR (Integrated stress response) inhibitor (ISRIB), which has been shown to reverse the effects of eIF2α phosphorylation to reduce ATF4 expression. When added to OSCC cells treated with phenformin, ISRIB decreased the expression of ATF4, DDIT4, NIBAN1, and autophagic markers that were induced by phenformin (Fig. 9a and Fig. S11c, d). Moreover, GSK2606414, a PERK inhibitor, efficiently inhibited PERK activation and decreased the phosphorylation of eIF2α. The induced expression of ATF4 together with DDIT4, NIBAN1, and autophagic markers elicited by phenformin treatment was also suppressed by the addition of the PERK inhibitor GSK2606414 (Fig. 9b and Fig. S11e). Having demonstrated that the induction of autophagy by phenformin could promote apoptosis (Fig. 3g), we performed Western blot analysis of apoptotic markers in phenformin-treated cells in the presence of the PERK inhibitor ISRIB, and the results showed that the PERK inhibitor could significantly block the apoptotic activity induced by phenformin (Fig. 9c and Fig. S11f). Finally, both the PERK inhibitor and the ISR inhibitor could partially rescue the growth inhibition of phenformin on OSCC cells (Fig. 9d). Taken together, we conclude that phenformin induces ER stress to directly activate the translation machinery to increase the expression of ATF4, which controls DDIT4 and NIBAN1 to promote autophagy formation, which leads to an increase in cell apoptosis that causes the suppression of OSCC cell growth (shown schematically in Fig. 9e).

## Discussion

Phenformin has been demonstrated to have more potent anti-tumor activities than metformin in different tumor types including melanoma, breast, colon, lung, and prostate cancers.^41,42^ Consistent with that previous research, we found that phenformin suppresses OSCC cell growth more potently than metformin in vitro, and inhibits OSCC tumor growth more dramatically in vivo. The effects of phenformin on cancer cell growth have been reported to be mainly through inhibition of the complex I of the mitochondrial respiratory chain to activate AMPK and block the mTOR pathway, which impacts protein synthesis, DNA damage, cell cycle arrest, blocking proliferation, and cell survival.^41,42^ Here, we found that phenformin not only inhibits OSCC cell proliferation, but also promotes apoptosis and autophagy to suppress OSCC cell growth in vivo and in vitro. Interestingly, RNA-seq data analysis revealed that the top enrichment pathway regulated by phenformin in OSCC cells was the autophagy-related pathway, which was significantly increased by phenformin treatment, hence we focused on characterizing the effect of phenformin on OSCC cell autophagy and its underlying mechanism.

Autophagy is a self-digestive process that helps maintain homeostasis by promoting the renewal of proteins and organelles, and an increased level of autophagy is a cell-survival mechanism in stressed cells.^43^ Due to their higher metabolic demands, rapid proliferation, and strong stress,^44^ cancer cells may require more autophagy for their survival.^45^ Although it has been proven that suppressing autophagy can augment the treatment effects of various cancer therapies,^46^ the induction of autophagic cell death can efficiently kill certain types of cancer cells, and is well recognized as a promising strategy to treat various types of cancers including OSCCs.^10,43,44,46^ It is not surprising that biguanide drugs are expected to trigger autophagy since both metformin and phenformin induce AMPK activation which results in the inhibition of mTOR, one of the major regulators of autophagy.^47^ Metformin has been reported to promote autophagic cell death to play an anti-tumor function in many types of cancer cells, including gastric cancer cells, ovarian cancer SKOV3 cells, and hepatocellular carcinoma cells.^48–50^ Phenformin was reported to enhance apoptosis and autophagy to suppress cell growth,^51^ though few studies have investigated the effects of phenformin on the regulation of cancer cell autophagy. Here, we found that phenformin treatment markedly increased the expression of the autophagy-related genes *ATG5* and *ATG7*, the lysosome marker Lamp-1 and the autophagosome markers LC3 and p62 in CAL 27 and SCC-9 cells, which shows that phenformin can clearly induce autophagy in OSCC cells. Our further study characterizing the effects of phenformin on autophagic flux revealed that phenformin promotes OSCC cell autophagy primarily by inducing autophagic formation without blocking autophagic flux (Fig. 3a–e). The knockdown of the autophagic gene *Beclin-1* could suppress increased apoptotic activity, as well as the inhibition of cell growth by phenformin. We anticipated that phenformin suppresses OSCC cell growth through the regulation of multiple pathways, which may explain why the knockdown of Beclin-1 couldn’t fully rescue the growth inhibition effect of phenformin. It has been reported that the accumulation of autophagosomes directly induces cellular toxicity^52^ and our results show that phenformin-induced cellular autophagy promotes apoptosis and plays a cytotoxic role in the inhibition of OSCC cell growth.

Phenformin-induced cellular autophagy was further supported by enrichment pathway analysis of RNA-seq data, which identified DDIT4 and NIBAN1 as two new major downstream targets of phenformin. Phenformin strongly enhanced the expression of DDIT4 and NIBAN1 in OSCC cells both in vivo and in vitro, and importantly, the knockdown of either DDIT4 or NIBAN1 suppressed the expression of autophagic markers induced by phenformin. This suggests that phenformin induces OSCC cell autophagy depending on DDIT4 and NIBAN1 expression. DDIT4 (DNA damage inducible transcript 4), also known as DNA damage response 1 (REDD1), can be induced by cellular stress conditions such as hypoxia or ER stress and starvation.^53–56^ As a negative regulator of mTOR, DDIT4 has been found to regulate multiple cellular functions such as growth, proliferation, and autophagy. DDIT4 has also been reported to promote autophagosome initiation and formation, as well as increasing autophagic flux through inhibition of the mTOR pathway in rat ventricular myocytes,^57^ indirectly supporting that phenformin enhances autophagic formation in OSCC cells. The *NIBAN1* (niban apoptosis regulator 1) gene, also known as *FAM129A*, belongs to the FAM129 family (family with sequence similarity 129), which has important cellular functions in a number of diseases and carcinogenic processes.^58^ NIBAN1 plays a significant role in cellular homeostasis through the regulation of cell stress and autophagy.^35,58,59^ In this study, we show that phenformin treatment strongly increased the expression of DDIT4 and NIBAN1 in OSCC cells in vivo and in vitro, and importantly, the knockdown of either DDIT4 or NIBAN1 suppressed the expression of autophagic markers induced by phenformin, which suggests that phenformin induces OSCC cell autophagy dependent on DDIT4 and NIBAN1 expression.

A previous study by Zhang et al. reported that the expression of DDIT4 was increased significantly along with activated autophagy induced by oxygen and energy deprivation in HT22 cells (a neuronal line of mouse hippocampus), and could be further increased by treatment with the AMPK activator AICAR.^60^ Therefore, we speculated that phenformin, another AMPK activator, induced DDIT4 and/or NIBAN1 through the activation of AMPK in OSCC cells. However, the inhibition of AMPK expression by the siRNA mediated knockdown of AMPK α1 and α2 did not block the increased expression of DDIT4 or NIBAN1 in phenformin-treated OSCC cells, indicating that phenformin promotes DDIT4 and NIBAN1 expression in an AMPK-independent manner. This finding was unexpected, but not completely surprising, since biguanides have previously been reported to inhibit the mTORC1 signaling pathway either dependent or independent of AMPK activation.^41,42^ For example, metformin independent of AMPK was reported to inhibit mTORC1 in a rag GTPase-dependent manner.^61^ Importantly, metformin was shown to suppress the mTOR pathway by the induction of REDD1 (DDIT4) expression in H1299 NSCLC cells, which was through the regulation of ATF4 expression.^34^ Our previous study reported that phenformin could induce ATF3 expression in melanoma cells.^62^ Here we observed that phenformin could induce both ATF3 and ATF4 expression in OSCC cells. The knockdown of ATF4, but not ATF3, could block the phenformin-induced expression of DDIT4 and NIBAN1, which suggested that phenformin regulates DDIT4 and NIBAN1 expression through the regulation of ATF4. The identification of DDIT4 and NIBAN1 as two new targets of phenformin that contribute to the promotion of autophagic cell death in OSCCs holds significant promise for the exploitation of new therapeutic approaches. This discovery opens avenues for potential interventions that target DDIT4 and NIBAN1, offering a promising new direction for future OSCC treatments.

*ATF4* belongs to the stress response ATF gene family, is induced by different cellular stresses and is involved in a program of gene expression patterns related to nutrient uptake, metabolism, and anti-oxidation.^63^ Central to early events in stress response pathways is a family of protein kinases, such as PKR-like ER kinase (PERK), that phosphorylate the subunit of eukaryotic initiation factor-2 (eIF2) following which the phosphorylated eIF2 (eIF2-P) inhibits global translation consistent with the preferential translation of ATF4.^63,64^ PERK is an ER protein that regulates signal transduction during ER stress,^65^ and the PERK/eIF2α/ATF4 pathway has been shown to play an important role in stress-induced autophagy gene expression.^66^ A previous study reported that serum deprivation induces ER stress to activate PERK, which results in enhanced eIF2α phosphorylation to induce ATF4 expression leading to facilitated transcription of the *REDD1* gene in Rat 2 fibroblasts.^67^ We also found that phenformin treatment induces ER stress to activate the PERK/eIF2-P/ATF4 axis pathway to control DDIT4 and NIBAN1 expression in OSCC cells. Importantly, PERK inhibitors were found to rescue the increased apoptotic activities and the inhibition of cell growth elicited by phenformin treatment, which suggests that phenformin may induce ER stress to directly promote apoptotic and autophagic cell death independent of AMPK activation, suggesting that ER stress plays a crucial role in cell death induced by phenformin in OSCC cells. ER stress activates unfolded protein response (UPR) pathways through the increased protein kinase PERK activation, which results in the elevation of eIF2α, leading to enhancing a pro-adaptive signaling pathway by the global protein synthesis inhibition and selective translation of ATF4. As a result of prolonged ER stress, pro-adaptive responses are suppressed and autophagic and apoptotic cell death follow.^68^ The present study indicates that the phenformin targeting of ER stress pathways may be more effective in eliminating of OSCC cells and may potentially help develop more promising anti-OSCC therapeutic drugs. These findings highlight the therapeutic potential of phenformin in the context of OSCC treatment.

In summary, the present study identifies two new phenformin targets, DDIT4 and especially NIBAN1, as novel targets of biguanides that have not been previously reported. Here we report that independent of AMPK activation, phenformin induces ER stress to activate the PERK/eIF2α/ATF4 axis pathway to enhance DDIT4 and NIBAN1 expression, which results in mTOR inhibition to promote autophagy to suppress OSCC cell growth (shown schematically in Fig. 9e). Phenformin combined with BRAF and MEK inhibitors is currently undergoing a clinical trial for the treatment of BRAF-mutated melanoma (NCT03026517). Our study further supports the anti-cancer function of phenformin and provides a solid basis to facilitate the development of phenformin for the clinical treatment of OSCC in the future.

## Materials and methods

### Reagents

Phenformin (Sigma, Santa Fe, NM, USA) and metformin (Sigma) stock solutions were prepared with phosphate buffered saline (PBS) for in vitro and in vivo studies.^29^ Chloroquine (CQ), MHY1485, ISRIB, and GSK2606414 were obtained from MedChemExpress (Monmouth Junction, NJ, USA) and were diluted with DMSO for in vitro studies.

### In vitro cell culture

All OSCC cell lines were purchased from the American Type Culture Collection (Rockville, MD, USA). The OSCC cell lines SCC-4, SCC-25, and CAL 27 were maintained in Dulbecco’s Modified Eagle Medium basic (DMEM basic) (Gibco, Grand Island, NY, USA) supplemented with 10% fetal bovine serum (FBS) (Biological Industries, Beit Haemek, Israel), penicillin (100 IU/mL), and streptomycin (100 µg/mL) (Gibco). SCC-9 cells were cultured in Dulbecco’s Modified Eagle Medium: F-12 (DMEM/F-12) containing 10% FBS, 400 ng/mL hydrocortisone, penicillin (100 IU/mL), and streptomycin (100 µg/mL). All cells were cultured in a cell incubator at 37 °C in a humidified 5% CO_2_ atmosphere.

### Normal gingival epithelial cells isolation and culture

The procedure for isolating and culturing normal gingival epithelial cells (oral keratinocytes) basically followed our previous publication.^69^ K-SFM (10744019, Gibco, Grand Island, NY) containing penicillin (100 IU/mL) and streptomycin (100 µg/mL) was used to culture gingival epithelial cells, and passage 3 cells were used for the experiments.

### Cell counting kit-8 (CCK-8) assay

CCK-8 assays were used to analyze the numbers of viable OSCC cells or gingival epithelial cells treated with phenformin or metformin following a previously published protocol.^70^ Cells were seeded in 96-well plates at 5 000 cells per well and were grown overnight. Cells were cultured with the desired concentrations of phenformin or metformin as shown in the figures. At the assay time points shown in the figures, the medium was replaced with DMEM basic containing 10% CCK-8 working solution (Biosharp, Guangzhou, China). At 2 h, a multi-functional enzyme marking instrument (BMGLABTECH, Offenburg, Germany) was used to measure OD values at 450 nm.

### Ethynyl-2**′**-deoxyuridine (EdU) staining assay

An EdU Cell Proliferation Image Kit (Abbkine, Wuhan, China) was used for EdU staining assays following the manufacturer’s instructions. SCC-9 cells and CAL 27 cells were seeded on sterile round coverslips in 96-well plates with 5 000 cells per well. The next day, OSCC cells were treated with 1 mmol/L phenformin for 48 h and were then incubated with EdU solution for 2 h in a cell incubator. After removing the medium, 0.1 mL 4% formaldehyde was added to each well and incubated for 15 min at room temperature. The cells were then washed with BSA washing solution three times and treated with PBS with 0.5% Triton X-100 for 20 min, followed by washing with BSA washing solution and staining with Click-iT reaction mixture in the dark for 30 min. After washing the cells with BSA washing solution, one drop of DAPI dye was added to each well, after which the cells were observed using a fluorescence microscope (Olympus BX53-DP80, Tokyo, Japan).

### Western blot analysis

Cells were washed with ice-cold PBS and lysed with radioimmunoprecipitation assay buffer (Solarbio, Beijing, China) containing 1% phenylmethanesulfonyl fluoride (Beyotime, Shanghai, China) and a 1% phosphatase inhibitor cocktail (Selleckchem, Houston, TX, USA) for 5 min on ice. The extracted proteins were centrifuged at 12 000 r/min for 10 min at 4 °C, followed by collecting the supernatants. The concentration of protein in each sample was measured using a BCA Protein Assay Kit (Solarbio). The supernatants were then mixed with 5 × SDS–PAGE loading buffer (Beyotime) and denatured at 100 °C for 5 min. Equal amounts of protein samples were electrophoresed in 10% or 12% sodium dodecyl sulfate-polyacrylamide gels (Beyotime) and were then electrotransferred to 0.45 µm polyvinylidene fluoride membranes (Merck Millipore, Billerica, MA, USA). The membranes were then incubated with 5% BSA blocking buffer (Solarbio) for 1 h and a primary antibody at 4 °C overnight, with GAPDH used as an intrinsic reference protein. The next day, each membrane was washed with Tris-based saline-Tween-20 (TBST) buffer (Servicebio, Wuhan, China) three times for 10 min each and incubated with corresponding secondary antibodies for 1 h at room temperature, followed by washing with TBST buffer three times again. The protein bands were visualized with Chemiluminescent HRP Substrate (Merck Millipore) and observed using a Multi-functional Imaging System (Shenhua, Hangzhou, China).

The following primary and secondary antibodies were used: PARP Antibody (Cell Signaling Technology (CST), Cat.#9542, Boston, MA, USA), Caspase-3 Antibody (CST, Cat.#9662), Cleaved Caspase-3 (Asp175) (5A1E) Rabbit mAb (CST, Cat.#9664), Atg7 (D12B11) Rabbit mAb (CST, Cat.#8558), SQSTM1/p62 (D1Q5S) Rabbit mAb (CST, Cat.#39749), Beclin-1 (D40C5) Rabbit mAb (CST, Cat.#3495), LC3A/B Antibody (CST, Cat.#4108), Lamp1 antibody (CST, Cat.#9091), Recombinant Anti-REDD-1/DDIT4 antibody (Abcam, Cat.ab191871, Cambridge, UK), FAM129A Polyclonal antibody (Proteintech, Cat.No.21333-1-AP, Chicago, IL, USA), mTOR (7C10) Rabbit mAb (CST, Cat.#2983), Phospho-mTOR (Ser2448) (D9C2) XP Rabbit mAb (CST, Cat.#5536), p70 S6 Kinase (49D7) Rabbit mAb (CST, Cat.#2708), Phospho-p70 S6 Kinase (Thr389) Antibody (CST, Cat.#9205), 4E-BP1 (53H11) Rabbit mAb (CST, Cat.#9644), Phospho-4E-BP1 (Thr70) Antibody (CST, Cat.#9455), AMPKɑ (D5A2) Rabbit mAb (CST, Cat.#5831), Phospho-AMPKɑ (Thr172) (40H9) Rabbit mAb (CST, Cat.#2535), Atg5 Antibody (CST, Cat.#2630), ATF-3 (E9J4N) Rabbit mAb (CST, Cat.#18665), ATF-4 (D4B8) Rabbit mAb (CST, Cat.#11815), BiP (C50B12) Rabbit mAb (CST, Cat.#3177), CHOP (L63F7) Mouse mAb (CST, Cat.#2895), PERK (D11A8) Rabbit mAb (CST, Cat.#5683), Phospho-PERK/EIF2AK3 (Ser719) Polyclonal antibody (Proteintech, Cat.No.29546-1-AP), eIF2α (D7D3) XP^®^ Rabbit mAb (CST, Cat.#5324), Phospho-eIF2α (Ser51) (D9G8) XP^®^ Rabbit mAb (CST, Cat.#3398), GAPDH (D16H11) XP Rabbit mAb (CST, Cat.#5174), Goat anti-Rabbit IgG HRP (HUABIO, Cat.HA1001, Hangzhou, China) and Goat anti-Mouse IgG Antibody (H&L)[HRP] (GenScript, Cat.No.A00160, Piscataway, NJ, USA).

### Virus infection

AdV5-CMV-GFP-LC3 and GFP-RFP-LC3 were purchased from WZ Biosciences (Jinan, China) and viral infections were performed with the protocol described previously.^62^ In total, 2 × 10^5^ CAL 27 cells per well were plated in 6-well plates. The next day, the desired amount of virus (MOI = 100) was added to the growth medium without antibiotics. At 12 h, the cells were washed with PBS two times and maintained in growth medium for 24 h. The old medium was then replaced with serum-free medium or growth medium with or without phenformin according to the grouping. After treatment for 6 h, images were observed using a fluorescence microscope (Olympus BX53-DP80).

### RNA extraction and real-time quantitative reverse transcription PCR (qRT-PCR) assay

A *SteadyPure* Universal RNA Extraction Kit (Accurate Biology, Changsha, China) was used to extract the total RNAs of OSCC cells. The concentration of each total RNA was measured using a NanoDrop spectrophotometer (Thermo Fisher Scientific, Waltham, MA, USA). In total, 1 000 ng of each total RNA was reverse transcribed to complementary DNA using an *Evo M-MLV* RT Kit for qRCR (Accurate Biology). A SYBR Green Premix Pro Taq HS qPCR Kit (Accurate Biology) was used to perform PCR reactions with a LightCycler96 Instrument (Roche Diagnostics, Basel, Switzerland) following the manufacturer’s instructions and the PCR program was carried out at 95 °C for 30 s, followed by 45 cycles at 95 °C for 5 s and at 60 °C for 30 s and ended with an elongation step for 15 s at 72 °C. Relative mRNA expression levels were normalized to the mRNA expression level of GAPDH. The primers of all genes tested are shown in Table S1.

### Flow cytometry (FACS) analysis

The effect of phenformin on the apoptosis of OSCC cells was measured using an Annexin V, FITC Apoptosis Detection Kit (Dojindo, Kumamoto Prefecture, Japan) as described previously.^71^ Similarly, OSCC cells were plated in 6-well plates with 2 × 10^5^ cells per well. After incubation with phenformin for 24 h, those cells were collected and resuspended in ice-cold PBS. Apoptosis of the control group (Con) cells was induced by repeated freeze (−20 °C for 20 min)-thawing (37 °C for 3 min) three times. After centrifugation, 1 × 10^5^ cells from each group were resuspended in 500 μL 1 × binding buffer containing 5 μL Annexin V-FITC conjugate and/or 5 μL PI solution according to the grouping and was incubated in the dark for 15 min on ice. Apoptosis rates were analyzed using a flow cytometer (FACS Calibur, BD Biosciences, Franklin Lakes, NJ, USA).

### In vivo tumor xenograft assay

Eight-week-old female nude/nude mice were purchased from Beijing Vital River Laboratory Animal Technology Co. (Beijing, China) and were randomly divided into two groups of three mice each: a control group (Con, PBS) and an experimental group (Phen, 150 mg/kg/day phenformin). Procedures of tumor xenografts were as described previously.^72^ In brief, 1 × 10^6^ OSCC cells suspended in 100 μL DMEM-opti (Gibco) were injected subcutaneously into four sites in the dorsal skin of each nude mouse. At 1 week, the nude mice in the Phen group were fed with phenformin dissolved in PBS twice a day for 2 weeks, while mice in the control group were fed with an equal amount of PBS as a control. At 2 weeks, all mice were euthanized and weighed, and their tumors were collected, weighed, and photographed for analysis.

### Immunohistochemical (IHC) staining and immunofluorescence (IF) staining

IHC and IF staining were performed as previously described.^62^ The tumor tissues were cut into 5-micron sections. Sections on slides were deparaffinized by immersion in dewaxing solution, anhydrous ethanol, and different concentrations of alcohol, in that order and were subsequently immersed in EDTA antigen repair solution (Solarbio) for antigen repair. After blocking endogenous peroxidase in the sections using a 3% hydrogen peroxide solution, 3% BSA (Solarbio) was added to cover the tissue sections evenly at room temperature. The blocking solution was gently shaken off and the sections were incubated with each primary antibody in a humidified box at 4 °C overnight. After washing three times with PBS for 5 min, the sections were incubated with the corresponding secondary antibody and washed three times with PBS again. For IHC staining, freshly prepared DAB solution was added dropwise to the sections and hematoxylin was used to stain cell nuclei, followed by dehydration. For IF staining, sections were incubated with DAPI for 5 min in the dark to stain nuclei. An immunofluorescence microscope (Olympus BX53-DP80) was used for analysis of staining.

The following primary and secondary antibodies were used: Anti-Ki67 antibody (Abcam, Cat.ab16667), Anti-LC3B antibody (Abcam, Cat. ab232940), SQSTM1/p62 (D6M5X) Rabbit mAb (CST, Cat.#23214), Anti-Beclin 1 antibody (Abcam, Cat.ab62557), Recombinant Anti-REDD-1/DDIT4 antibody (Abcam, Cat.ab191871), FAM129A Polyclonal antibody (Proteintech, Cat.No.21333-1-AP), and DyLight 594 goat anti-rabbit IgG (H + L) (Multisciences, Cat.GAR007, Hangzhou, China).

### RNA-seq analysis

Total RNAs from CAL 27 and SCC-9 OSCC cells treated with phenformin or PBS (Control) for 12 h were collected and purified using a *SteadyPure* Universal RNA Extraction Kit. Purified RNAs were sent to Beijing Genomics Institute (BGI, Beijing, China) for RNA sequencing and further analysis. DEGs were screened with | log_2_ Fold Change | > 1.5 and *q* value < 0.05. Volcano plots, KEGG (Kyoto Encyclopedia of Genes and Genomes) Pathway Network Diagrams and heat maps were plotted based on DEGs.

### siRNA transfection

siRNA transfections were performed as described previously.^72^ Briefly, CAL 27 cells were plated in 6-well plates at 3 × 10^5^ cells per well. The next day, those CAL 27 cells were transfected with the specific siRNA or the scramble siRNA (Control) diluted in DMEM-opti containing Lipofectamine 3000 (Thermo Fisher Scientific). After 24 h, the transfected cells were treated with phenformin or with PBS (Control) for the desired times as indicated in the figures. The cells were then collected for qRT-PCR or Western blot analysis. The oligo sequences of siRNAs are listed in Table S2.

### Statistical analyses

All experiments were replicated three times. Data summarization included the mean ± standard deviation of at least three scoring results and statistical analysis was performed using GraphPad Prism 7. Student’s *t* test was used to compare two groups, one-way or two-way analysis of variance (ANOVA) was used to compare three or more groups. “*” indicates a significant difference between groups.

### Supplementary information

Supplementary Materials

## Data Availability

The datasets used and analyzed in this study are available from the corresponding author upon reasonable request.
