# Supplementary material for: Phenformin activates ER stress to promote autophagic cell death via NIBAN1 and DDIT4 in oral squamous cell carcinoma independent of AMPK
Source: Int J Oral Sci. 2024 May 8;16:35. doi: 10.1038/s41368-024-00297-w (PMC11079060; doi:10.1038/s41368-024-00297-w)
Supplement: Supplementary file 1 — Supplementary Materials [file 41368_2024_297_MOESM1_ESM.pdf]

Supporting Information

Phenformin activates ER stress to promote autophagic cell death via NIBAN1 and DDIT4 in oral squamous cell carcinoma independent of AMPK

Running Title: Phenformin induces OSCC autophagic cell death

Dexuan Zhuang[1, 2], Shuangshuang Wang[1], Huiting Deng[2], Yuxin Shi[2], Chang Liu[1], Xue Leng[1], Qun Zhang[1], Fuxiang Bai[1], Bin Zheng[3], Jing Guo[1,2*], Xunwei Wu[1, 2*]

[1]School and Hospital of Stomatology, Cheeloo College of Medicine, Shandong University & Shandong Key Laboratory of Oral Tissue Regeneration & Shandong Engineering Research Center of Dental Materials and Oral Tissue Regeneration & Shandong Provincial Clinical Research Center for Oral Diseases, No. 44-1 Wenhua Road West, Jinan, Shandong, China, 250102.

17854161152@163.com (D.Z.); 201915889@mail.sdu.edu.cn (S.W.); Lc15662655244@163.com (C.L.); 15163156576@163.com (X.L.); zhangqunpku@126.com (Q.Z.); 15866727236@163.com (F.B.); guojing@sdu.edu.cn (J.G.); xunwei_2006@hotmail.com (X.W.)

[2]Engineering Laboratory for Biomaterials and Tissue Regeneration, Ningbo Stomatology Hospital, Savaid Stomatology School, Hangzhou Medical College, Ningbo, Zhejiang, China, 315016.

881012021008@hmc.edu.cn (H.D.); 881012022069@hmc.edu.cn (Y.S.)

[3]Cedars-Sinai Cancer Institute, Department of Biomedical Sciences, Cedars-Sinai Medical Center, Los Angeles, CA, USA, 90069.

bin.zheng@cshs.org (B.Z.)

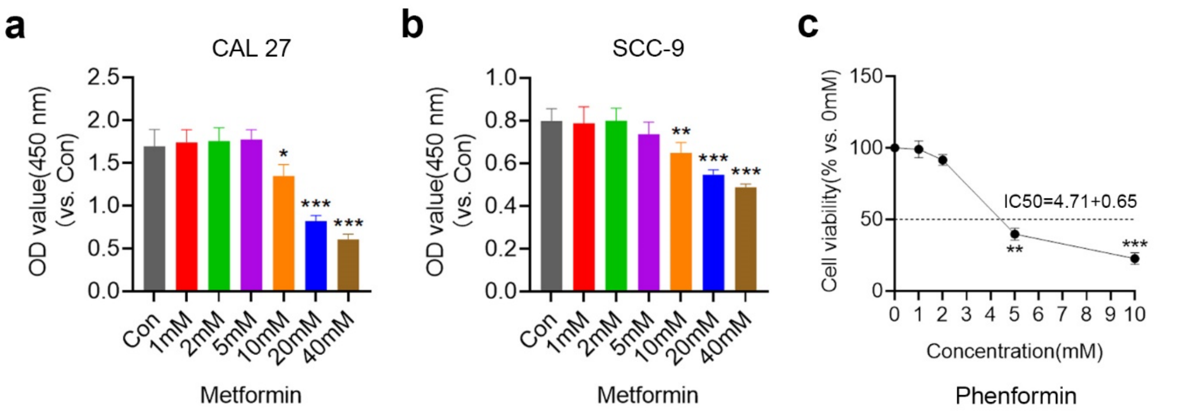

Figure S1. The role of biguanides on the growth of OSCC and normal gingival epithelial cells. **a, b** OSCC cell lines CAL 27 (**a**) and SCC-9 (**b**) were treated with different concentrations of metformin as indicated, with PBS used as a control. At 48 h, cells were collected and analyzed using the CCK-8 assay for cell viability. **c** Normal gingival epithelial cells were treated with various concentrations of phenformin as indicated. At 48 h, cells were collected and analyzed using the CCK-8 assay for cell viability. All experiments were repeated for 3 times, error bars represent means ± SD in each group; P values are indicated with "*", * indicates P<0.05, ** indicates P<0.01, *** indicates P<0.001.

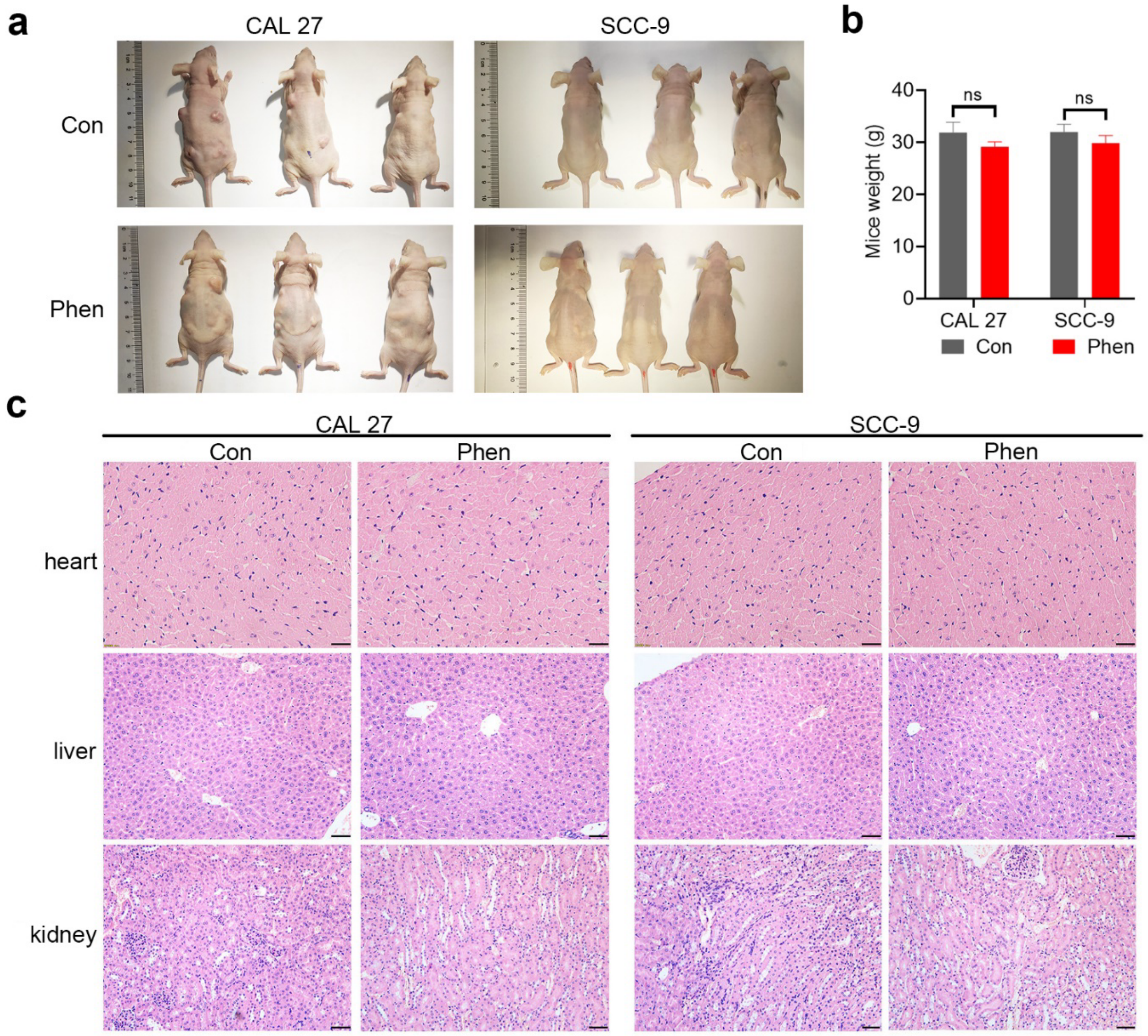

Figure S2. Treatment with phenformin reduces tumor size but doesn't significantly affect nude mouse health. **a** Images of mice after xenografting CAL 27 (left) and SCC-9 (right) cells following oral administration of either phenformin (150 mg/kg) or PBS (control) for 2 weeks. **b** Quantitation of body weights of mice treated with phenformin or PBS. **c** Images of H&E staining of heart, liver and kidney tissues from mice fed with phenformin or PBS for 2 weeks. Scale bars = 20 µm.

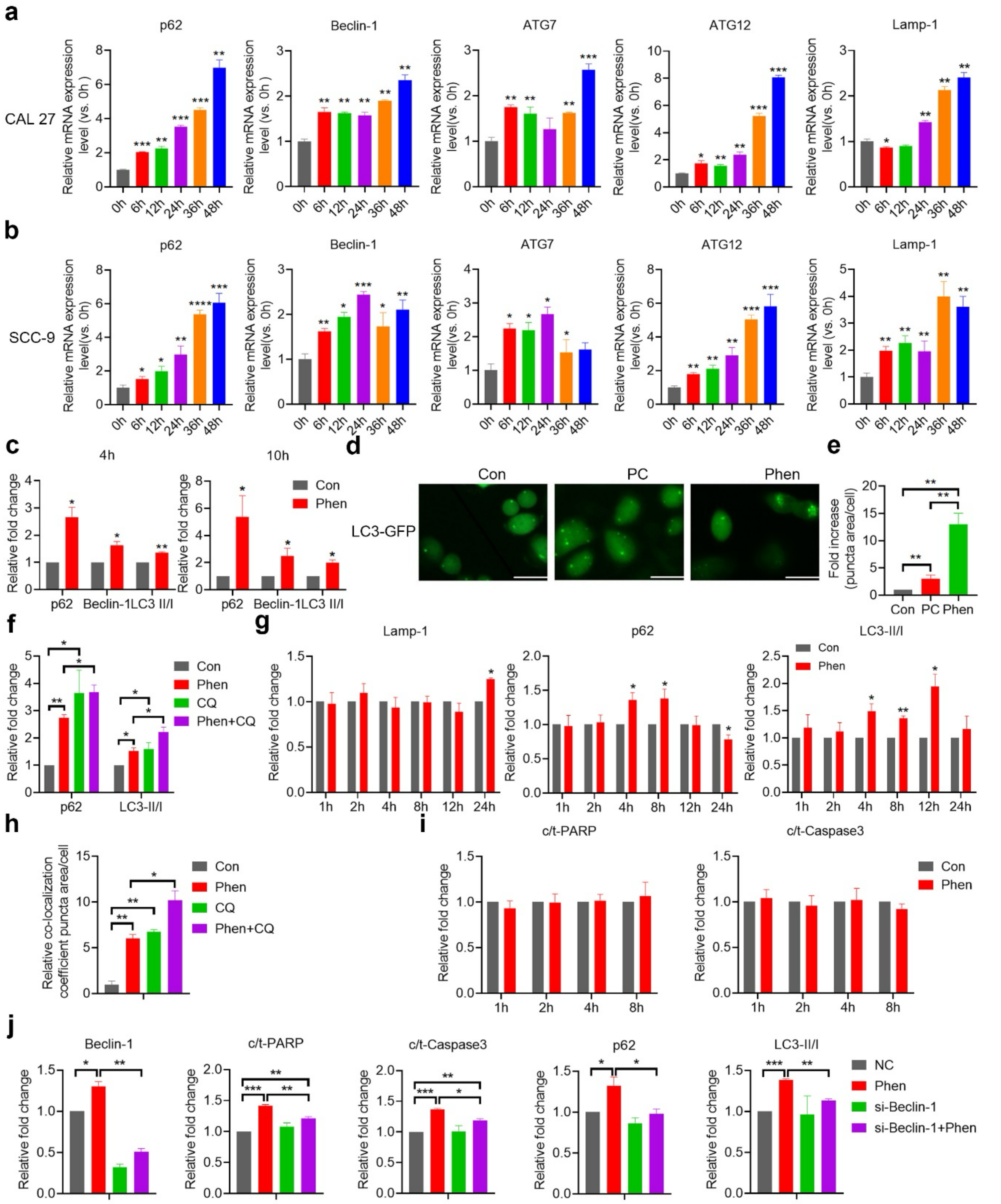

Figure S3. Phenformin promotes OSCC autophagy. **a**,**b** Relative mRNA expression levels of autophagy markers (*p62*, *Beclin-1*, *ATG7*, *ATG12* and *Lamp-1*) normalized by *GAPDH* in CAL 27 cells (**a**) and in SCC-9 cells (**b**) treated with or without 1 mM phenformin at 6, 12, 24, 36 and 48 h analyzed by qRT-PCR. **c** Quantification of the relative protein levels of p62 and Beclin-1 in Fig. 3a normalized to the GAPDH band; the relative expression level of the LC3-II was normalized to LC3-I band. **d** low magnification image of Fig. 3b, Scale bars = 20 μm. **e** Puncta area per cell in (**d**) was calculated using Image J software. **f** Quantification of the relative protein levels of p62 in Fig. 3c normalized to the GAPDH band; the relative expression level of the LC3-II was normalized to LC3-I band. **g**

Quantification of the relative protein levels of Lamp-1 and p62 in Fig. 3d normalized to the GAPDH band; the relative expression level of the LC3-II was normalized to LC3-I band. **h** Relative co-localization coefficient puncta area per cell in Fig. 3e was calculated using Image J software. **i** Quantification of the relative protein levels of c-PARP and c-Caspase3 in Fig. 3f normalized to the corresponding total protein band. **j** Quantification of the relative protein levels of Beclin-1 and p62 in Fig. 3g normalized to the GAPDH band; the relative expression level of c-PARP and c-Caspase3 was normalized to the corresponding total protein and LC3-II was normalized to LC3-I band. All experiments were repeated for 3 times, error bars represent means ± SD in each group; P values are indicated with "*", * indicates P<0.05, ** indicates P<0.01, *** indicates P<0.001, **** indicates P<0.0001.

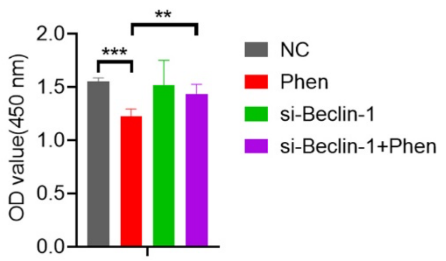

Figure S4. Inhibition of autophagy reduces the growth inhibitory effect of phenformin on OSCC cells. CCK-8 analysis of CAL 27 cells transfected with scramble siRNAs or Beclin1 siRNAs plus/minus 1 mM phenformin at 24 h and incubated with 1 mM phenformin or PBS as a control for 24 h. The experiments were repeated for 3 times, error bars represent means ± SD in each group; P values are indicated with "*", ** indicates P<0.01, *** indicates P<0.001.

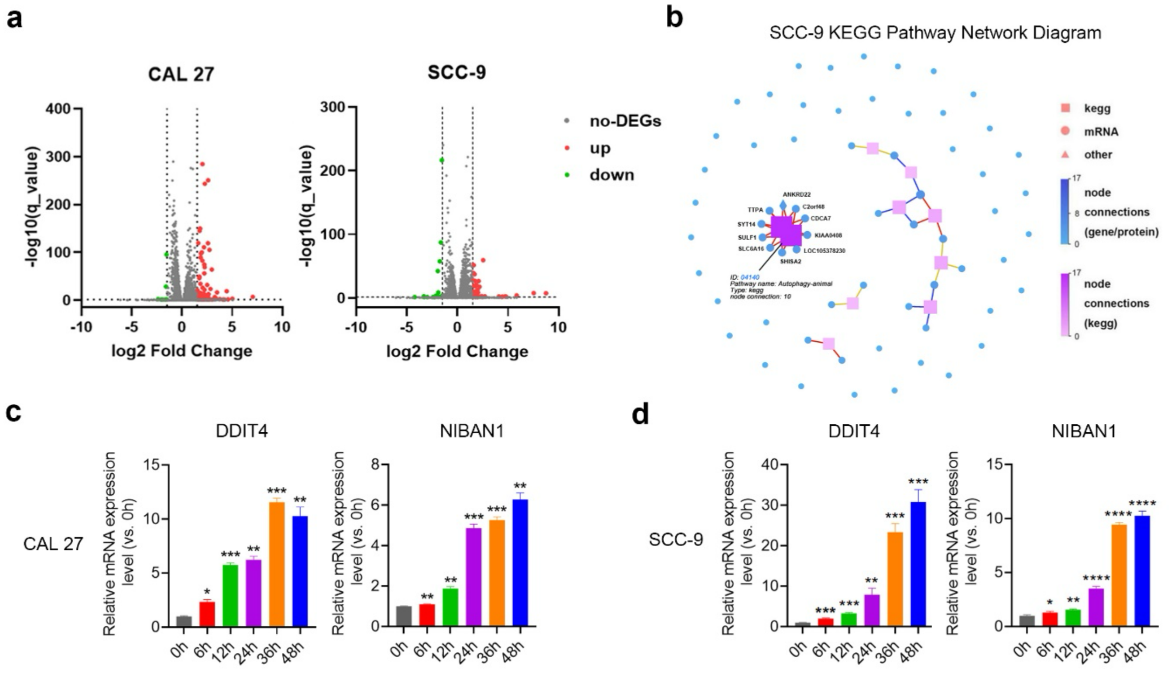

Figure S5. Phenformin regulates the autophagic signaling pathway and induces expression of the autophagy-related genes DDIT4 and NIBAN1. **a** Volcano plot visualizing DEGs of CAL 27 (left) and

SCC-9 cells (right) between the Phen group and the control group at 12 h. The q value <0.05 and |log2 Fold change| >1.5 were used as a threshold to determine the significance of DEGs. Red dots represent up-regulated DEGs, blue dots represent down-regulated DEGs, and gray dots indicate transcripts that did not change significantly between the two groups. **b** KEGG Pathway Network Diagram of DEGs in SCC-9 cells. Blue circles and red squares represent different mRNAs and KEGG pathways, respectively. The darker the color represents the more mRNAs or KEGG pathway were connected to the pathway or mRNA. **c, d** Relative expression levels of DDIT4 and NIBAN1 normalized by GAPDH in CAL 27 cells (**c**) and SCC-9 cells (**d**) treated with or without 1 mM phenformin at 6, 12, 24, 36 and 48 h analyzed by qRT-PCR. The experiments of **c**, **d** were repeated for 3 times; P values are indicated with "*", * indicates P<0.05, ** indicates P<0.01, *** indicates P<0.001, **** indicates P<0.0001.

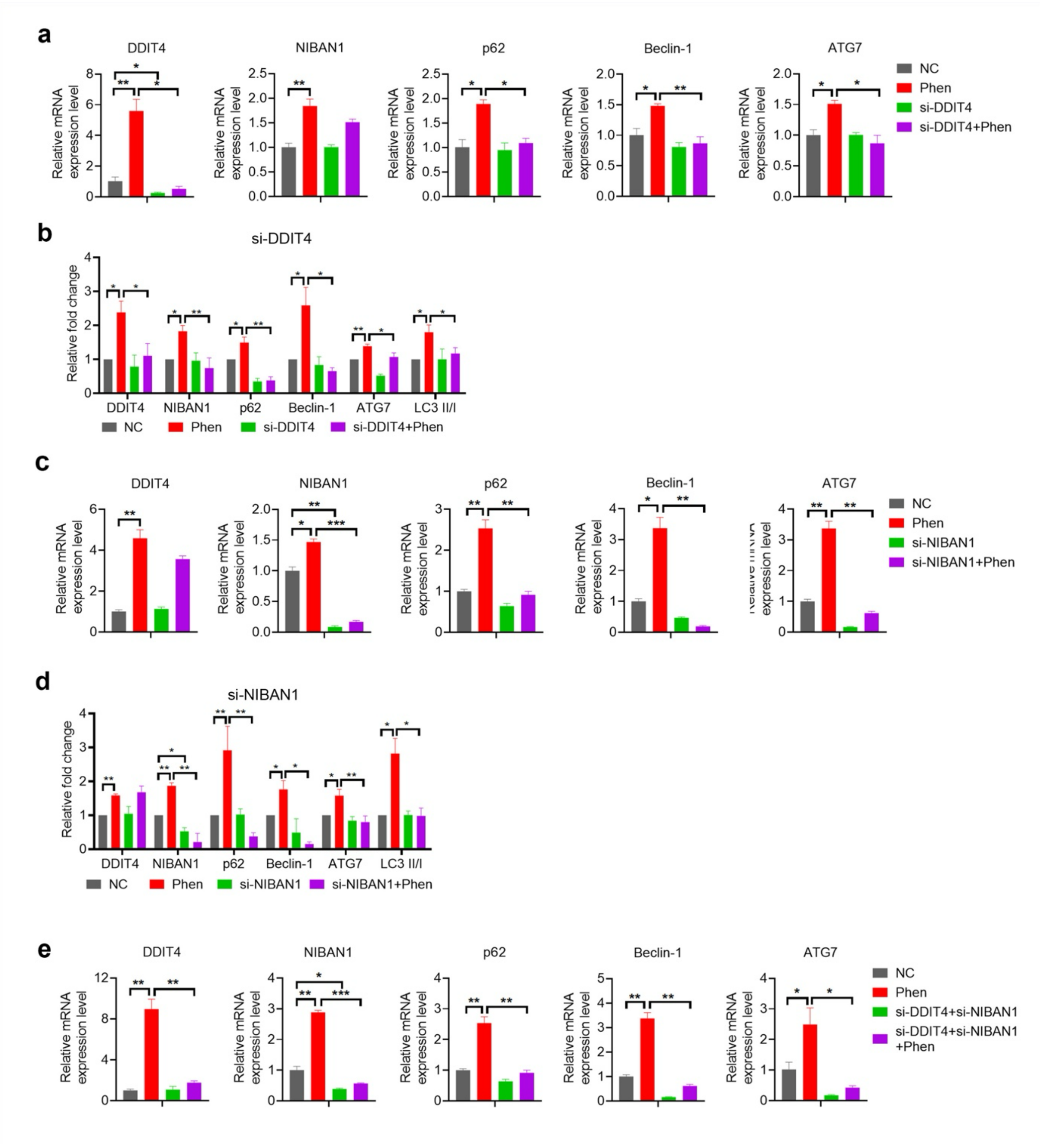

Figure S6. Inhibition of DDIT4 or NIBAN1 and double inhibition of DDIT4 and NIBAN1 expression

block phenformin-induced autophagy. **a,** Expression of *DDIT4*, *NIBAN1*, *p62*, *Beclin-1* and *ATG7* analyzed by qRT-PCR in CAL 27 cells transfected with si-DDIT4 or the corresponding controls (NC) at 12 h after treatment with 1 mM phenformin (Phen) or with PBS as a control. **b** Quantification of the expression levels of p62, Beclin-1, ATG7, DDIT4 and NIBAN1 proteins in Fig. 6a was normalized to the GAPDH band, while the expression level of LC3-II in Fig. 6a was normalized to LC3-I. **c,** Expression of *DDIT4*, *NIBAN1*, *p62*, *Beclin-1* and *ATG7* analyzed by qRT-PCR in CAL 27 cells transfected with si-NIBAN1 or the corresponding controls (NC) at 12 h after treatment with 1 mM phenformin (Phen) or with PBS as a control. **d,** Quantification of the expression levels of p62, Beclin-1, ATG7, DDIT4 and NIBAN1 proteins in Fig. 6b was normalized to the GAPDH band, while the expression level of LC3-II (in Fig. 6a, b) was normalized to LC3-I. (**b**) Expression of *DDIT4*, *NIBAN1*, *p62*, *Beclin-1* and *ATG7* analyzed by qRT-PCR in CAL 27 cells transfected with both si-DDIT4 and si-NIBAN1 (or the corresponding controls (NC) at 12 h after treatment with 1 mM phenformin (Phen) or with PBS as a control. All experiments were repeated for 3 times, error bars represent means ± SD in each group; P values are indicated with "*", * indicates P<0.05, ** indicates P<0.01, *** indicates P<0.001.

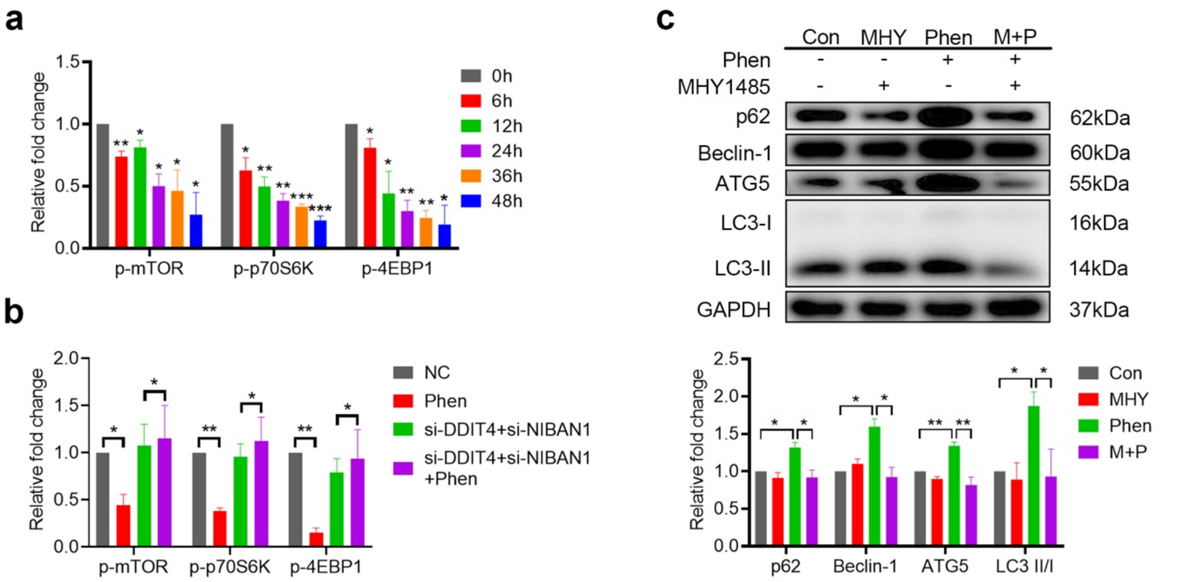

Figure S7. Phenformin inhibits mTOR phosphorylation to promote autophagy by regulating DDIT4 and NIBAN1 expression in OSCC cells. **a** Quantification of the relative levels of p-mTOR, p-p70S6K and p-4EBP1 proteins (in Fig. 6c) normalized to the corresponding total protein band. **b** Quantification of the relative levels of p-mTOR, p-p70S6K and p-4EBP1 proteins (in Fig. 6d) normalized to the corresponding total protein band. **c** Upper: Immunoblotting analysis of the autophagy markers p62,

Beclin-1, ATG5 and LC3-I/II in CAL 27 cells treated with DMSO (Con), phenformin (1 mM) with or without MHY1485 (10 μM) for 12 h. GAPDH is used as a loading control. Lower: Quantification of the relative levels of p62, Beclin-1 and ATG5 in was normalized to the GAPDH band, the LC3-II protein band was normalized to the LC3-I protein band. All experiments were repeated for 3 times, error bars represent means ± SD in each group; P values are indicated with "*", * indicates P<0.05, ** indicates P<0.01, *** indicates P<0.001.

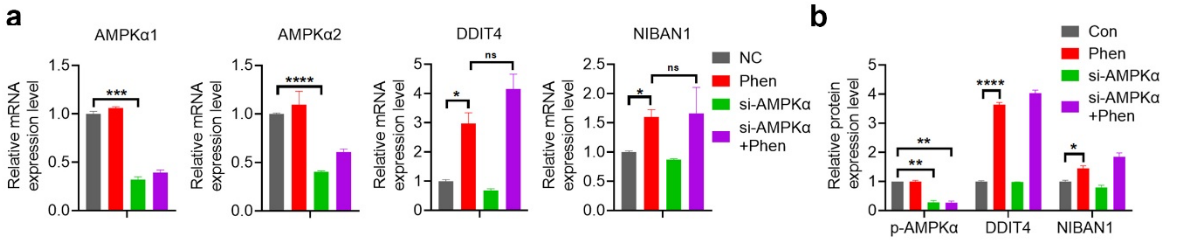

Figure S8. Expression of DDIT4 and NIBAN1 is not regulated by AMPK in OSCC cells. **a** Expression levels of AMPKα (AMPKα1 and AMPKα2), DDIT4 and NIBAN1 analyzed by qRT-PCR in CAL 27 cells transfected with AMPKα1 siRNA plus AMPKα2 siRNA or with the corresponding controls (NC) at 12 h after treatment with 1 mM phenformin (Phen) or with PBS as a control. **b** Quantification of the relative levels of p-AMPKα, DDIT4 and NIBAN1 (in Fig. 7a) were normalized to the AMPKα and GAPDH bands, respectively. Error bars represent means ± SD in each group; P values are indicated with "*", * indicates P<0.05, ** indicates P<0.01, *** indicates P<0.001, **** indicates P<0.0001.

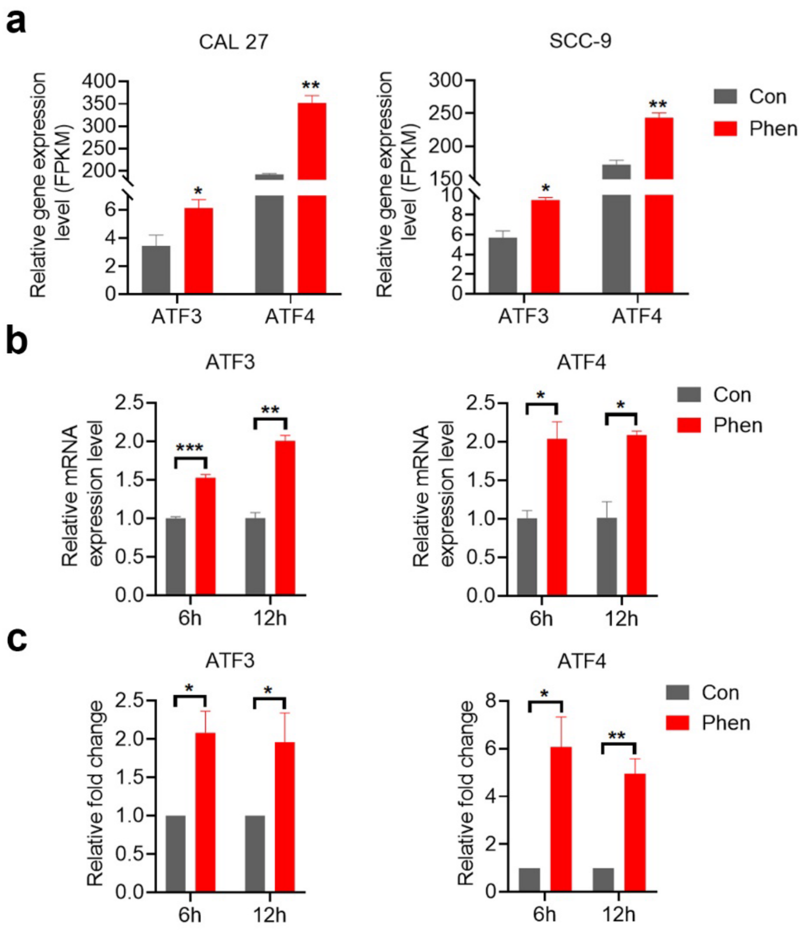

Figure S9. RNA-seq analysis and qRT-PCR show that ATF3 and ATF4 expression is promoted by phenformin treatment. **a** ATF3 and ATF4 expression levels (FPKM) were measured in CAL 27 and SCC-9 cells treated with phenformin (Phen) or with PBS (Con) for 12 h. **b** Expression of ATF3 and ATF4 analyzed by qRT-PCR in CAL 27 cells at 6 and 12 h after treatment with 1 mM phenformin (Phen) or with PBS as a control. **c** Quantification of the relative levels of ATF3 and ATF4 normalized to the GAPDH band (in Fig. 7b). All experiments were repeated for 3 times, error bars represent means ± SD in each group; P values are indicated with "*", * indicates P<0.05, ** indicates P<0.01, *** indicates P<0.001.

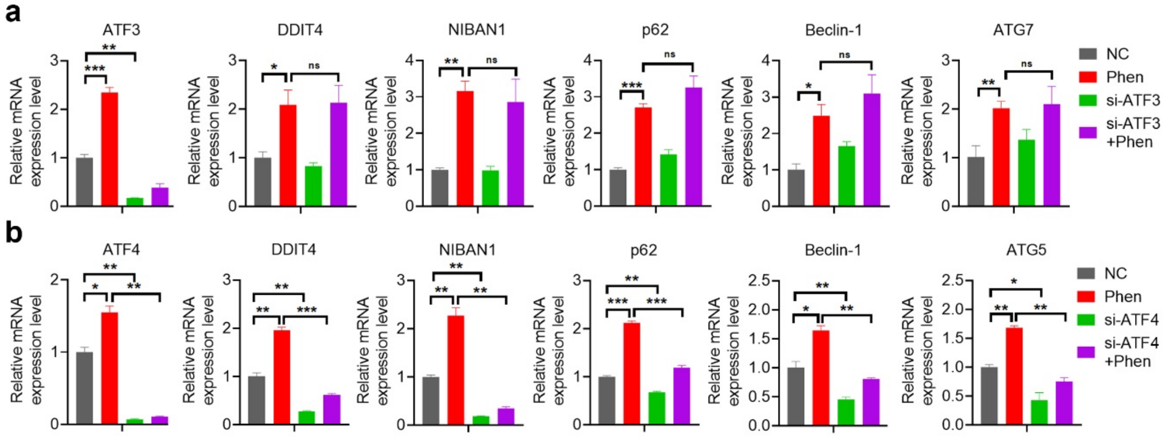

Figure S10. Phenformin regulates DDIT4 and NIBAN1 expression by inducing ATF4 expression, but not ATF3. **a** Expression of ATF3, DDIT4, NIBAN1, p62, Beclin-1 and ATG7 analyzed by qRT-PCR in CAL 27 cells transfected with ATF3 siRNA (si-ATF3) or corresponding controls (NC) at 12 h after treatment with 1 mM phenformin (Phen) or with PBS as a control. **b** Expression of ATF4, DDIT4, NIBAN1, p62, Beclin-1 and ATG5 analyzed by qRT-PCR in CAL 27 cells transfected with ATF4 siRNA (si-ATF4) or corresponding controls (NC) at 12 h after treatment with 1 mM phenformin (Phen) or with PBS as a control. All experiments were repeated for 3 times, error bars represent means ± SD in each group; P values are indicated with "*", * indicates P<0.05, ** indicates P<0.01, *** indicates P<0.001.

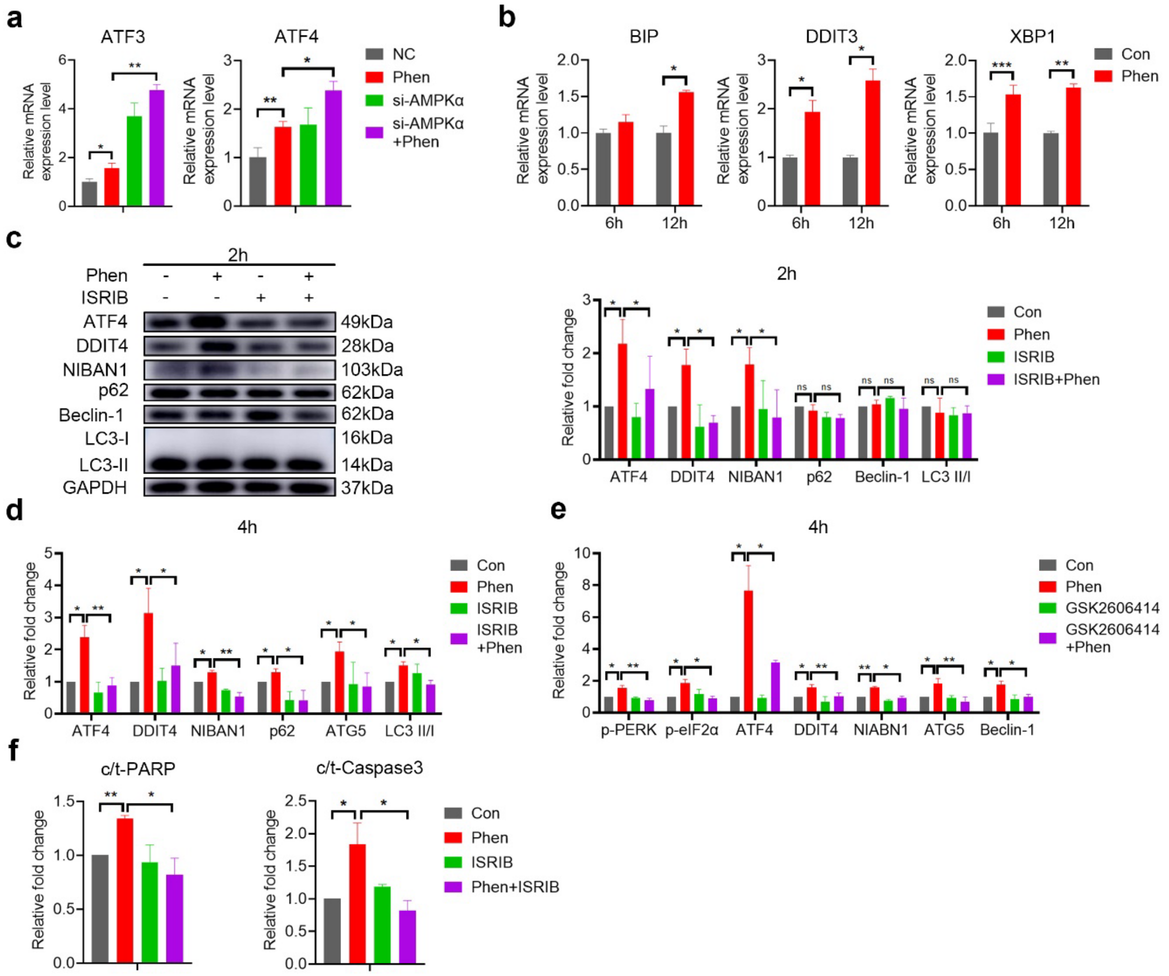

Figure S11. Phenformin induces the expression of ATF4, DDIT4 and NIBAN1 through the regulation of ER stress. **a** Expression levels of ATF3 and ATF4 analyzed by qRT-PCR in CAL 27 cells transfected with AMPKα1 siRNA and AMPKα2 siRNA or corresponding controls (NC) at 12 h after treatment with 1 mM phenformin (Phen) or with PBS as a control. **b** Expression of BIP, DDIT3 and XBP1 analyzed by qRT-PCR in CAL 27 cells at 6 and 12 h after treatment with 1 mM phenformin (Phen) or with PBS as a control. **c** Left: Protein expression levels of ATF4, DDIT4, NIBAN1, p62, Beclin-1 and LC3-I/II analyzed by Western blot in CAL 27 cells at 2 h after treatment with 1 mM phenformin (Phen) or 200 nM ISRIB or DMSO as a control; Right: Quantification of LC3-II was normalized to LC3-I and quantification of the other protein bands was normalized to GAPDH. **d** Quantification of LC3-II (in Fig. 9a) was normalized to LC3-I and quantification of the other protein bands (in Fig. 9a) was normalized to GAPDH. **e** Quantification of ATF4,

DDIT4, NIBAN1, ATG5 and Beclin-1 protein (in Fig. 9b) was normalized to GAPDH and quantification of p-PERK and p-eIF2α protein (in Fig. 9b) was normalized to the corresponding total protein bands. **f** Quantification of the relative levels of c-PARP and c-Caspase 3 (in Fig. 9c) normalized to the corresponding total protein bands. All experiments were repeated for 3 times, error bars represent means ± SD in each group; P values are indicated with "*", * indicates $P<0.05$, ** indicates $P<0.01$, *** indicates $P<0.001$.

Table S1. Oligo sequences of primers used in the present study.

Gene		Oligo sequence（5'-3'）
GAPDH	Forward	CTCCTCCGGGTGATGCTTTT
	Reverse	ATGAAGGGGTCATTGATGGCA
p62	Forward	GGATCCGAGTGTGAATTTCC
	Reverse	CTCTGTGCTGGAACTCTCT
Beclin-1	Forward	CAGGAACTCACAGCTCCATT
	Reverse	CATCAGATGCCTCCCCAATC
ATG5	Forward	CTGCACTGTCCATCTAAGG
	Reverse	AGTTTCCGATTGATGGCC
ATG7	Forward	CAGATGGAGTAGCAGTTTCC
	Reverse	GGTCCATACATTCACTGAGG
ATG12	Forward	CTGGCGACACCAAGAAGAAA
	Reverse	GGATGGTTCTTGTTCGCTCTAC
Lamp-1	Forward	CTTTCAAGGTGGAAGGTGGC
	Reverse	GATAGTCTGGTAGCCTGCGT
DDIT4	Forward	CTTGTGTGCCAACCTGAT
	Reverse	AGGCGCAGTAGTTCTTTG
NIBAN1	Forward	CTCAGCCCTTTGTGGTCCT
	Reverse	CTCCTGTCGGAAGAATTGCAC
AMPKα1	Forward	GCACCTTCGGCAAAGTGAAG
	Reverse	CCTACCACATCAAGGCTCCG
AMPKα2	Forward	TCTGTAAGCATGGACGGGTT
	Reverse	AGATGACTTCAGGTGCTGCA
ATF3	Forward	GTCCATCACAAAAGCCGAGG
	Reverse	GCACTCCGTCTTCTCCTTCT
ATF4	Forward	GCCAAGCACTTCAAACCTCA
	Reverse	GGTCATCTGGCATGGTTTCC
BIP	Forward	CTGGGTACATTTGATCTGACTGG
	Reverse	GCATCCTGGTGGCTTTCCAGCCATTC
DDIT3	Forward	CCACTCTTGACCCTGCTTCT
	Reverse	TGGTTCTCCCTTGGTCTTCC
XBP1	Forward	GGAGCTGGGTATCTCAAATC
	Reverse	GTTTACACCAAGCAGAGAGG

Table S2. Oligo sequences of siRNAs used in the present study.

siRNA		Oligo sequence（5’-3’）
Negative control（NC）	Forward	UUCUCCGAACGUGUCACGUTT
	Reverse	ACGUGACACGUUCGGAGAATT
DDIT4-Homo-833	Forward	GCUUCCGAGUCAUCAAGAATT
	Reverse	UUCUUGAUGACUCGGAAGCTT
DDIT4-Homo-UTR-1160	Forward	GUAGCAUGUACCUUAUUAUTT
	Reverse	AUAAUAAGGUACAUGCUACTT
DDIT4-Homo-UTR-1315	Forward	GGAGGUGGUUUGUGUAUCUTT
	Reverse	AGAUACACAAACCACCUCCTT
NIBAN1-Homo-1972	Forward	CCAGCUUAACAGAUCUAAATT
	Reverse	UUUAGAUCUGUUAAGCUGGTT
PRKAA1-Homo-330	Forward	GAGGAGAGCUAUUUGAUUATT
	Reverse	UAAUCAAAUAGCUCUCCUCTT
PRKAA2-Homo-1032	Forward	GCAGUGGCUUAUCAUCUUATT
	Reverse	UAAGAUGAUAAGCCACUGCTT
Homo-ATF3	Forward	GGAAAGUGUGAAUGCUGAATT
	Reverse	UUCAGCAUUCACACUUUCCTT
Homo-ATF4	Forward	AGAAGAUGGUAGCAGCAAATT
	Reverse	UUUGCUGCUACCAUCUUCUTT
Beclin-1-Homo-991	Forward	CAGUUUGGCACAAUCAAUATT
	Reverse	UAUUGAUUGUGCCAAACUGTT
